# Green-engineered agricultural-based nano-adsorbent for efficient Cr(vi) removal: batch mechanisms and continuous column performance

**DOI:** 10.1039/d5ra09397j

**Published:** 2026-01-09

**Authors:** Archana Kushwaha, Zeenat Arif, Bineeta Singh, Ranjeet Kumar Mishra

**Affiliations:** a Department of Chemical Engineering, Harcourt Butler Technical University Kanpur 208002 India dariflt@hbtu.ac.in; b Department of Chemical Engineering, Manipal Institute of Technology, Manipal Academy of Higher Education Manipal Karnataka 576104 India ranjeet.mishra@manipal.edu

## Abstract

This study presents a green, cost-effective, and sustainable strategy for remediation of Cr(vi) from surface water. The approach employs a bio-based nano-adsorbent derived from watermelon leaves, integrated with green-synthesised TiO_2_ nanoparticles using *Cajanus cajan* leaf extract. The acid-modified nano-adsorbent (A-WML/TiO_2_) demonstrated enhanced adsorption efficiency compared to pristine A-WML due to increased surface area (98.25 m^2^ g^−1^), abundant active functional groups, and synergistic adsorption–photocatalytic reduction properties. Furthermore, the batch experiments confirmed the optimum removal conditions to be pH 4, 25 mg of adsorbent dosage, 10 ppm of Cr(vi), and a contact time of 3 h, achieving a 94.23% removal. Additionally, photocatalytic studies under sunlight further reduced Cr(vi) to Cr(iii) with an efficiency of 81%. Kinetic analysis followed pseudo-second-order behaviour (*R*^2^ = 0.998), indicating chemisorption. In contrast, the Freundlich and Langmuir isotherm models confirmed heterogeneous multilayer adsorption with a predominant monolayer interaction for NA (*R*^2^ = 0.9815). Reusability tests revealed excellent stability after five regeneration cycles using NaOH with minimal loss in performance, highlighting the sustainability of materials. The fixed-bed column studies using nano-adsorbent-modified sand showed a markedly prolonged breakthrough time, increasing from 400 to 800 min. This improvement was achieved with a 3 wt% TiO_2_ loading at a 15 cm bed height under acidic pH, confirming the material's suitability for practical continuous water treatment systems. Thomas and Adams–Bohart model fitting confirmed the mass-transfer-controlled adsorption processes and predictive accuracy for column performance. A comparative evaluation with existing bio-adsorbents highlights a superior Cr(vi) removal capacity, dual functionality (adsorption and photocatalysis), and effective integration into sand filtration systems, thus enabling scalable deployment.

## Abstract 2

This study presents a green, cost-effective, and sustainable strategy for remediation of Cr(vi) from surface water.

## Graphical abstract

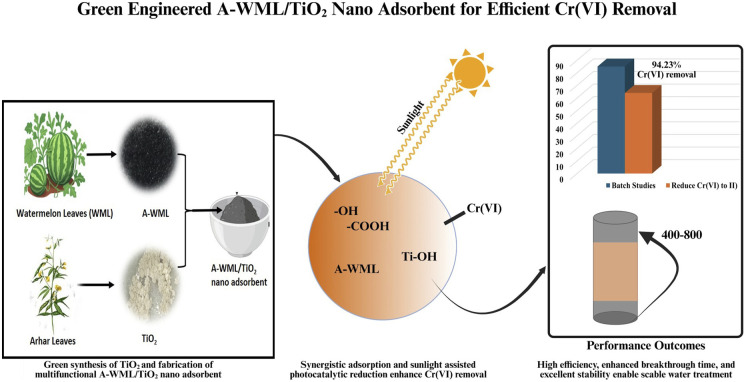

## 1. Introduction

Wastewater is defined as water contaminated by various human activities, rendering it unsuitable for its intended use. The release of toxic organic and inorganic pollutants from domestic, industrial, and other anthropogenic sources disrupts the environmental balance and threatens the entire ecosystem. Prolonged exposure to these contaminants poses a significant threat to human health and can cause irreversible damage to flora and fauna.^1^ Among inorganic pollutants, heavy metals (HMs) require special attention because of their ability to impair vital organs and drastically reduce energy levels. Hexavalent chromium (Cr(vi)), commonly discharged from industries such as tanning, electroplating, dyeing, and textiles, is of particular concern due to its high carcinogenicity and its potential to cause skin irritation and lung damage.^2^ Due to its persistence and tendency to bioaccumulate, chromium ranks fifth among 269 hazardous substances. Regulatory bodies such as the World Health Organisation (WHO) and the European Commission have set stringent permissible limits for Cr(vi) in water at 0.05 mg L^−1^ and 0.025 mg L^−1^, respectively.^3^ Although established remediation techniques, including membrane filtration, precipitation, coagulation, solvent extraction, ion exchange, and electro-dialysis, can remove contaminants, their widespread application is constrained by high operational costs and the risk of generating secondary pollutants or recontaminating the environment.^4^ In contrast, adsorption has gained significant attention due to its simplicity, adaptability, and energy efficiency. Recently, the valorisation of agricultural waste as an adsorbent has emerged as a sustainable and cost-effective approach. Many agro-wastes are rich in cellulose, hemicellulose, and lignin, containing functional groups such as hydroxyl, carboxyl, phenolic, and methoxy groups that facilitate the removal of Cr(vi).^5^ However, the relatively low removal efficiency of unmodified bio-adsorbents has shifted research efforts toward developing modified materials. Advances in materials science have enabled the transformation of traditional agro-based adsorbents into nano-engineered forms, enhancing surface area and porosity, thereby improving adsorption performance through the incorporation of nanoparticles (NPs).

Several studies highlight the potential of such modified adsorbents. Ref. 6 reported maximum adsorption capacities for Cd(ii), Cu(ii), Ni(ii), and Pb(ii) using chitosan–titanium dioxide (Cs–TiO_2_) composites, while Mahmoud & Ibrahim, 2023  successfully synthesised Ti-MOF@TiO_2_@WMPB@CTH bio-nanocomposites with strong affinity toward doxorubicin and Cr(vi).^7^ Ref. 8 demonstrated that chitosan–TiO_2_-coated sand achieved 96.76% and 98.91% removal of BOD and COD, respectively. Prabu *et al.*, 2022 utilised magnetic nano-adsorbents impregnated with activated carbon from animal bone waste to achieve a Cr(vi) adsorption capacity of 27.86 mg g^−1^.^9^ Campos *et al.*, 2019 reported that smaller core–shell bi-magnetic nano-adsorbents demonstrated nearly 40% higher adsorption capacity due to increased surface area.^10^ These materials have also been applied in fixed-bed continuous systems. For instance, ref. 11 used graphene oxide-enriched sand and showed that breakthrough curves for As(iii) followed the Adams–Bohart model, while other pollutants were best described by the Thomas model. Similarly, ref. 12 reported that xanthan gum/kaolinite modified with *Araucaria* gum efficiently removed atrazine, with breakthrough curves well-fitted to the Yoon–Nelson and Thomas models. Collectively, these studies highlight the potential of upgrading agro-based adsorbents with NPs to enhance adsorption performance.

Various waste biomasses have been successfully employed as adsorbents, including watermelon rinds/shells,^13,14^ sunflower biomass,^15^*Gossypium hirsutum*,^16^ and moringa leaves.^17^ However, despite their abundance and compositional richness, watermelon leaves (WML) have not yet been explored as an adsorbent. India, being the world's third-largest watermelon producer, generates large volumes of dried leaves as waste. Rich in amines (from proteins), hydroxyl groups (from cellulose), and carboxyl groups, WML offers a promising, low-cost bio-adsorbent for Cr(vi) removal. Nevertheless, the major drawback of using bare WML is the need for post-treatment to desorb accumulated Cr ions, which increases operational costs. This limitation can be addressed by integrating TiO_2_ nanoparticles into the adsorbent matrix, thereby imparting self-cleaning (photocatalytic) properties and enhancing adsorption performance. Following green chemistry principles, TiO_2_ nanoparticles in this study are synthesised *via* an eco-friendly route using the extract of Arhar (*Cajanus cajan*) leaves, a widely available pulse enriched with flavonoids, aromatic compounds, terpenoids, and amines that serve as natural reducing and stabilising agents. Embedding these TiO_2_ NPs into WML enables a dual-function mechanism, adsorption and photocatalytic reduction, within a single WML/TiO_2_ composite. This synergy facilitates both the capture of Cr(vi) ions and their reduction to the less toxic Cr(iii) form. The efficiency of the developed nano-adsorbent is further evaluated from batch experiments to a sand-mixed fixed-bed column system for heavy metal immobilisation. Various fixed-bed kinetic models are applied to simulate breakthrough behaviour and assess dynamic adsorption performance.

To date, no comprehensive study has investigated a bi-functional, waste-derived, green nano-adsorbent supported by both batch and column evaluations. This work addresses the existing gap and contributes to sustainable environmental management. This study directly supports key Sustainable Development Goals by enabling the safe and sustainable remediation of Cr(vi). Removing a highly toxic carcinogen from water enhances human health and well-being (SDG 3) while providing an effective and low-cost solution for access to clean water (SDG 6). The sunlight-driven photocatalytic pathway reduces energy demand, aligning with the United Nations' Sustainable Development Goals (SDG 7). Green synthesis using biomass waste lowers environmental impact and supports climate action (SDG 13). Furthermore, preventing Cr(vi) release safeguards aquatic ecosystems (SDG 14) and reduces contamination of soils and terrestrial life, protecting biodiversity on land (SDG 15).

## 2. Materials and methods

### 2.1 Sample collections and preparation

Watermelon leaves (WML) were sourced from the floodplain region of the Ganga River near the Ganga Barrage in Kanpur, Uttar Pradesh, India, for the preparation of the bio-adsorbent. The collected watermelon leaves (WML) were initially washed thoroughly with distilled water to remove surface impurities, such as dust. The cleaned leaves were then sun-dried (1–2 days), ground into fine powder, and sieved to obtain uniform particle sizes suitable for adsorbent preparation. Furthermore, the green synthesis of nanoparticles (NPs) involved the preparation of a separate plant extract using fresh Arhar (*Cajanus cajan*) leaves. These leaves were obtained from a nearby agricultural field and thoroughly washed several times with distilled water to ensure complete removal of dust and surface contaminants. The cleaned leaves were air-dried and used for preparing the aqueous extract. This dual-source approach ensured the use of locally available, renewable biomass materials for adsorbent preparation and nanoparticle biosynthesis.

### 2.2 Chemicals

*Tetraisopropyl orthotitanate* (TTIP) was procured from Sigma-Aldrich, which is used as he primary precursor for nanoparticle synthesis. Additional analytical-grade chemicals, including sulfuric acid (H_2_SO_4_) and methanol (SD Fine-Chem Ltd), sodium hydroxide (NaOH), 1,5-diphenylcarbazide (DPC), and potassium dichromate (K_2_Cr_2_O_7_) were purchased from Merck India Pvt. Ltd, India. Sterilised coarse sand (0.02–2 mm) required for filtration-related experiments was obtained from Vikas Sales Corporation, India. All reagents were of high purity (99%) and used without further purification.

### 2.3 Nano-adsorbent preparation

**Fig. 1:**
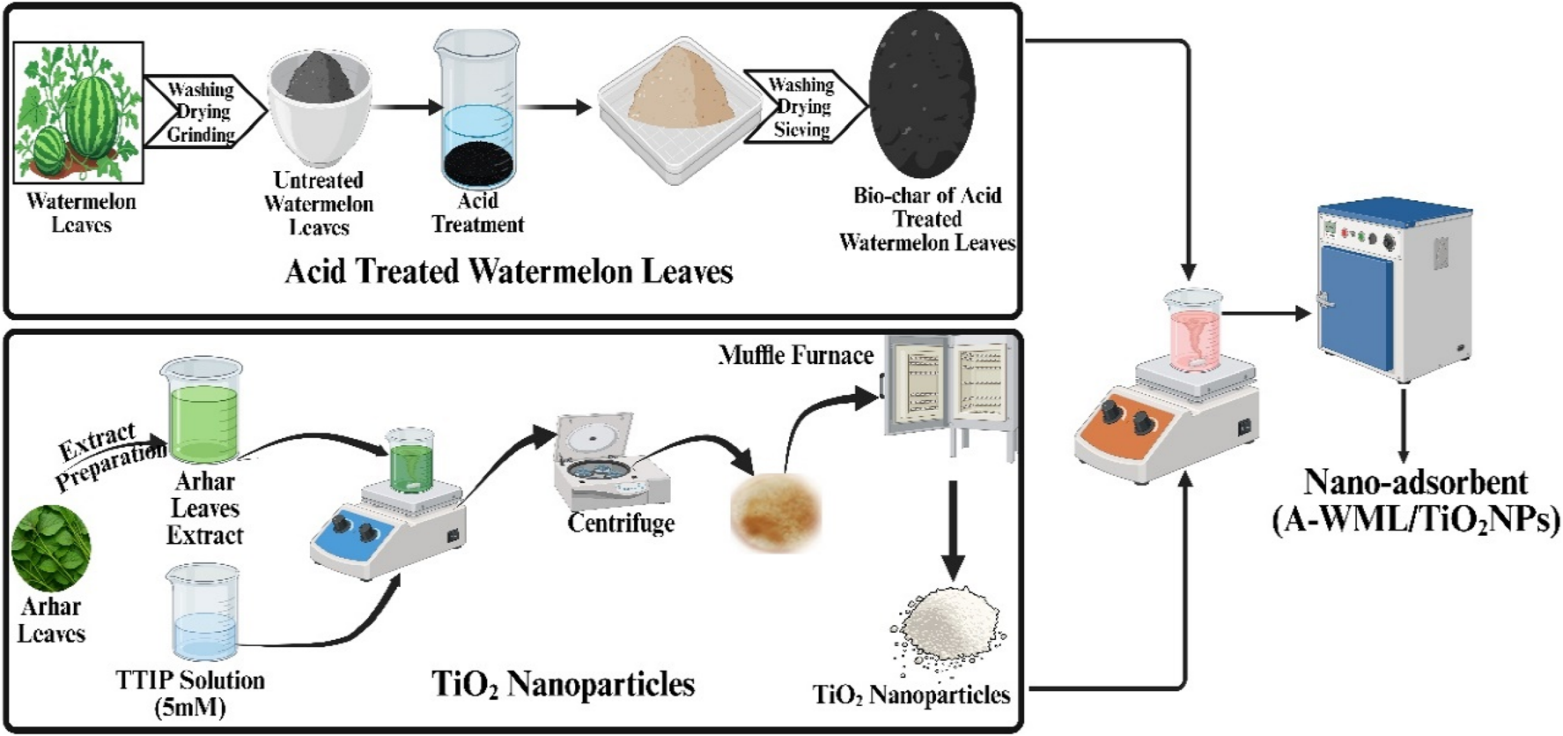
Schematic representation of the preparation of a nano-adsorbent for pollutant removal.

The bio-adsorbent (100 g) derived from watermelon leaves was first subjected to chemical activation using 0.1 M H_2_SO_4_, whereby the mixture was stirred for 30 min at ambient conditions to enhance its surface functionality and adsorption efficiency. Following acid treatment, the material was thoroughly rinsed to remove residual acid, dried, and designated as acid-modified watermelon leaf adsorbent (A-WML). Furthermore, the green synthesis of TiO_2_ nanoparticles (NPs) involved immersing 60 g of thoroughly washed Arhar dal (*Cajanus cajan*) leaves in 1 L of distilled water and heating for 60 min to extract phytochemicals known to act as natural reducing, stabilising, and capping agents. The resulting extract was filtered and mixed with an equal volume of 20 mM TTIP precursor in a 1 : 1 ratio. The reaction mixture was continuously stirred for 8 h, facilitating TTIP hydrolysis and TiO_2_ nucleation, which ultimately formed a stable nanoparticle suspension. Furthermore, the suspension was centrifuged, and the obtained precipitate was dried in a hot air oven at 105 °C for 1 h to yield fine TiO_2_ NPs. To impart photocatalytic functionality to the adsorbent, a quantified amount of TiO_2_ nano-powder was added to 100 mL of an A-WML slurry prepared using distilled water. The mixture was stirred at room temperature for 30 minutes, allowing for uniform deposition and surface attachment of TiO_2_ NPs. After filtration, the composite material was dried at 50 °C for 1 h, resulting in the final A-WML/TiO_2_ nano-adsorbent. A schematic representation of the overall preparation procedure is shown in Fig. 1. For experimental evaluation, two adsorbent types were employed: (i) pristine acid-modified adsorbent (PA = A-WML without TiO_2_) and (ii) nano-enhanced adsorbent (NA = A-WML loaded with 2 wt% of TiO_2_ nanoparticles per g adsorbent).

### 2.4 Characterisation of NPs and bio-adsorbent

The successful incorporation of TiO_2_ NPs in the adsorbent matrix, prepared using Arhar dal extract (ADE), was confirmed through X-ray diffraction (XRD) analysis performed on a Panalytical instrument equipped with Cu Kα radiation (*λ* = 1.5444 Å). Detection of active sites/functional groups responsible for adsorption of moieties was achieved through a Fourier transform infrared spectrophotometer (FTIR: Model (Spectrum 2) PerkinElmer Pvt. Ltd). The surface morphology of the nanoparticles and the synthesised adsorbents was examined using Field Emission Scanning Electron Microscopy (FESEM; SUPRA 40 VP). The specific surface area and porosity of the adsorbents were measured using the Brunauer–Emmett–Teller (BET) method (Autosorb IQ system, Chemisorption, TCD) at IIT Kanpur, employing the nitrogen absorption and desorption technique. A UV-Visible spectrophotometer (SHIMADZU CORP 80511, HBTU Kanpur) operating within the wavelength range of 190–800 nm was used to quantify Cr(vi) absorbance during adsorption experiments. Additionally, the surface charge characteristics of the adsorbent and nanoparticles were determined using a Zeta Potential Analyser (Model SZ-100, HORIBA Scientific, IIT Kanpur).

### 2.5 Batch adsorption experiment

Batch adsorption experiments were performed in two separate beakers (500 mL) to evaluate the Cr(vi) removal efficiency of the PA and NA nano-adsorbents. A fixed adsorbent dosage of 0.4 g L^−1^ was added to a synthetic Cr(vi) solution, and the suspension was continuously agitated on a magnetic stirrer at 500 rpm while maintaining a temperature of 30 °C. During each run, 10 mL aliquots were withdrawn at 5 min intervals to monitor the progress of adsorption. The collected samples were centrifuged, and the resulting supernatant was reacted with 0.25 mL of 1,5-diphenylcarbazide (DPC) reagent. The Cr(vi) concentration was then quantified by measuring absorbance at 560 nm using a UV-Vis spectrophotometer (Shimadzu Corp., Model 80511). Adsorption capacity (*q*_e_) and percentage were calculated based on eqn (1) and (2)^18^ presented in Table 1. Furthermore, to assess photocatalytic performance, identical experimental conditions were employed, but the studies were conducted under natural sunlight irradiation. Sample aliquots were collected at predetermined time intervals, and the residual Cr(vi) concentration was measured using the UV-Vis method. The photocatalytic removal efficiency of PA and NA was then determined using eqn (3)^19^ in Table 1. This approach enabled a comparative evaluation of both adsorptive and photocatalytic pathways for the remediation of Cr(vi).

**Table 1:** Various analytical parameters and modelling approaches were employed to evaluate the performance of the synthesised adsorbent.^18,20,21^

Parameter/model	Mathematical equation	Nomenclature	Equation no.
Adsorption capacity	*Q*_c_ = ([Cr]_*t*=0_ − [Cr]_*t*=*t*_min__) × *V*/*m*	[Cr]: Cr(vi) concentration (mg L^−1^), *V*: sample volume (L), *m*: adsorbent amount (g), *Q*_c_: adsorption capacity, [Cr]_i_: initial concentration of Cr, [Cr]_f_: final concentration of Cr	(1)
% adsorption	([Cr]_*t*=0_ − [Cr]_*t*=*t*_min__/[Cr]_*t*=0_) × 100		(2)
% photocatalytic reduction	([Cr]_i_ − [Cr]_f_/[Cr]_i_) × 100		(3)

**Kinetic model**			
Pseudo 1^st^ order	ln(*q*_e_ − *q*_*t*_) = ln *q*_e_ − *K*_1_*t*	*K*_1_, *K*_2_: rate constant for pseudo 1^st^ and 2^nd^ order, *q*_e_ & *q*_*t*_: Cr(vi) adsorbed at equilibrium and time (*t*) in (mg g^−1^)	(4)
Pseudo 2^nd^ order	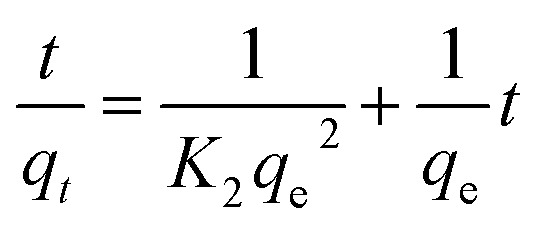		(5)

**Isotherm model**			
Langmuir	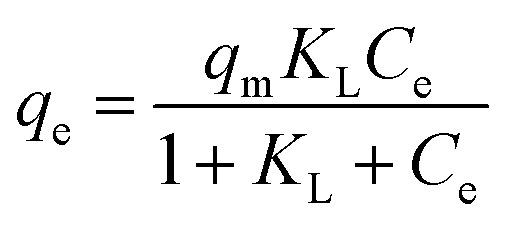	*q*_m_: maximum adsorption, *R*_L:_ separation factor, *K*_L_: Langmuir isotherm constant, *K*_f_: Freundlich isotherm constant (L mg^−1^), *K*_T_: Temkin isotherm constant, *n*: isotherm factor, *R*_T_, and *B*_T_: adsorption energy and binding constant	(6)
	*R*_L_ = 1/(1+ *K*_L_*C*_o_)		(7)
Freundlich	*q*_e_ = *K*_F_*C*_e_^1/*n*^		(8)
Temkin	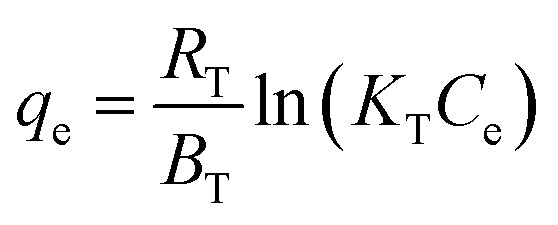		(9)

**Continuous column studies: kinetic model**			
Adams–Bohart	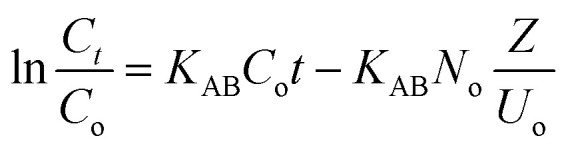	*K*_AB_ (L mg^−1^ min^−1^): Adams–Bohart const, *K*_TH_ (mL mg^−1^ min^−1^): Thomas const, *N*_0_ (mg L^−1^): volumetric adsorption capacity, *U*_0_ (cm min^−1^): velocity of solution: *Z* (cm): bed height, *m* (g): adsorbent mass, (mg L^−1^: Cr concentration at time *t*, (mg L^−1^): Cr concentration at time equal zero	(10)
Thomas	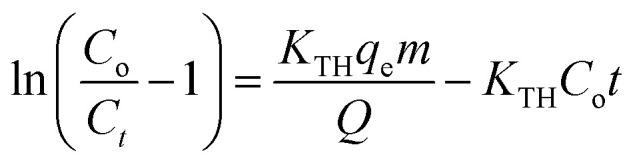		(11)

### 2.6 Adsorption kinetics and isotherm

Kinetic studies, including the pseudo-first-order and pseudo-second-order models (eqn (4) and (5) in Table 1)^22^ were employed to elucidate the underlying adsorption mechanism and to identify the rate-controlling step at the solid–solution interface.^23^ These models offer insights into whether the adsorption process is predominantly governed by physisorption or chemisorption. To further interpret the equilibrium behaviour of Cr(vi) adsorption onto the PA and NA adsorbents, three well-established isotherm models, such as Langmuir, Freundlich, and Temkin, were applied. The Langmuir isotherm assumes monolayer adsorption onto a homogeneous surface with uniform adsorption energies. The maximum monolayer adsorption capacity was estimated using eqn (6) (Kaur *et al.*, 2025; Priyadarsini *et al.*, 2024 in Table 1).^19,22^ From the linearised Langmuir plot, the constants *K*_L_ and *q*_m_ were obtained and subsequently used to calculate the separation factor (*R*_L_) *via* eqn (7).^22^ The *R*_L_ value serves as an indicator of adsorption favourability, where *R*_L_ > 1 denotes unfavourable adsorption, while 0 < *R*_L_ < 1 signifies a favourable and feasible adsorption process. In contrast, the Freundlich isotherm describes multilayer adsorption on a heterogeneous surface and is expressed by eqn (8).^20^ The Freundlich constants *K*_f_ and *n* were determined from the linear relationship between ln *q*_e_ and ln *C*_e_, offering insight into adsorption intensity and surface heterogeneity. Additionally, the Temkin isotherm model was applied to account for the effect of indirect adsorbate–adsorbent interactions. This model assumes that the heat of adsorption decreases linearly with increasing surface coverage. The Temkin constants were derived from the plot of *q*_e_*versus* ln *C*_e_, providing further insight into the energetic variations associated with the adsorption process.^18^

### 2.7 Desorption studies

Reusability and recyclability are critical indicators of the practical applicability and economic viability of any adsorbent material. To assess the potential of the synthesised green nano-adsorbents for repeated use in Cr(vi) removal, desorption and regeneration experiments were performed. After each adsorption cycle, the spent nano-adsorbent was treated with individual desorbing agents: 1 M NaOH, 1 M HNO_3_, and methanol by immersing it in the respective solutions for 120 min under gentle agitation, followed by filtration. The recovered solid was then thoroughly rinsed with deionised water to eliminate residual reagents and dried for 1 h to restore its functional state. The regenerated adsorbents were subsequently reintroduced into fresh Cr(vi) solutions using the same experimental protocol as in the initial adsorption run. This adsorption–desorption cycle was repeated five consecutive times to evaluate the long-term stability, structural integrity, and regeneration efficiency of the material. Monitoring the adsorption performance across successive cycles provided insight into possible degradation, loss of active sites, or structural alterations in the nano-adsorbent, thereby determining its suitability for sustainable water treatment applications.

### 2.8 Continuous (fixed-bed) column studies

**Fig. 2:**
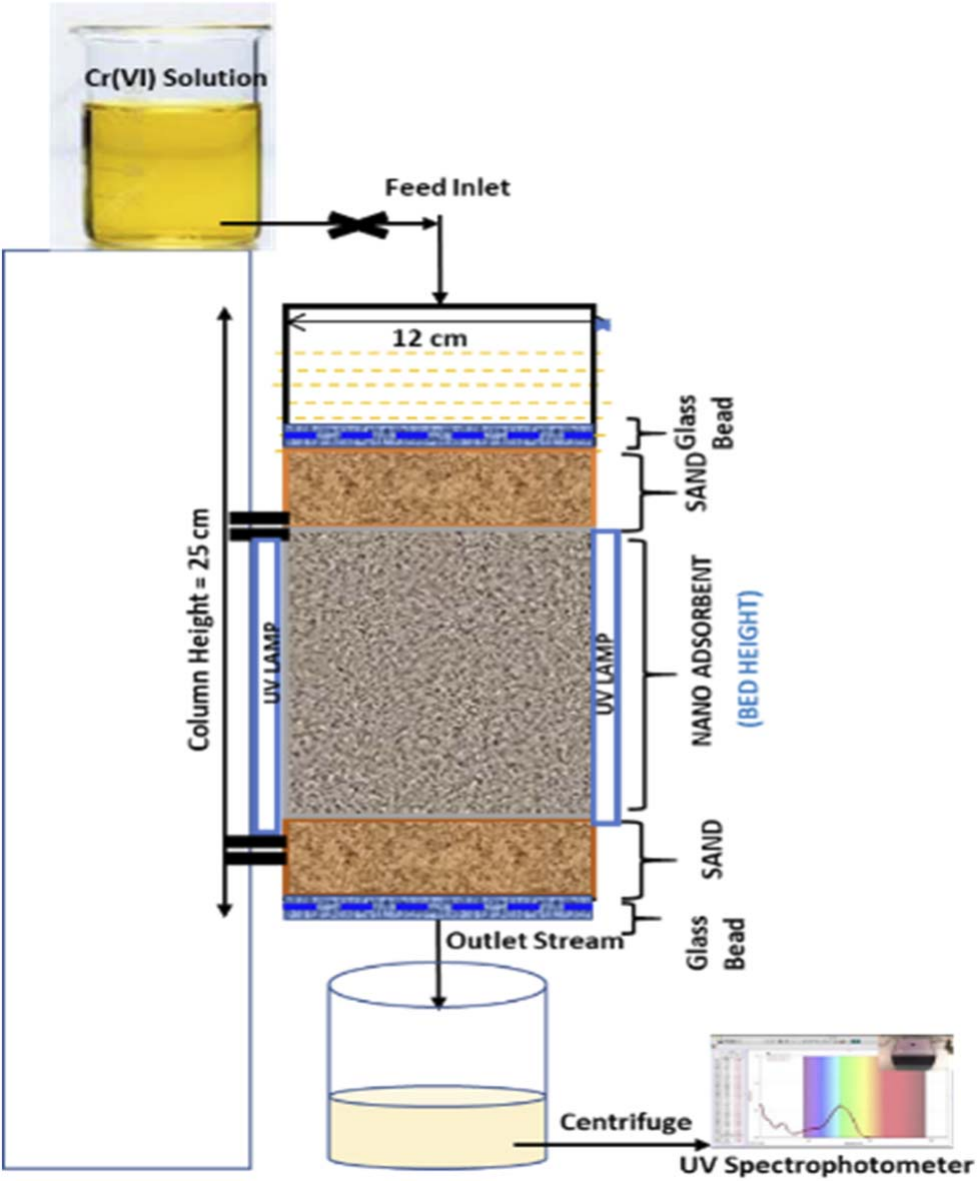
Schematic diagram of the continuous adsorption column.

In this study, continuous fixed-bed column experiments were performed using an NA/sand filtration system housed in a rectangular column measuring 12 × 12 × 25 cm (Fig. 2). To prevent channelling and ensure uniform flow distribution, the NA/sand filtration layer was placed between two 2 cm layers of porcelain glass beads, which also served as mechanical support for the packed bed. A schematic representation of the experimental setup and its essential components is provided in Fig. 2. The fixed-bed column experiments were designed to evaluate the breakthrough characteristics and overall performance of the NA/sand adsorbent under dynamic operating conditions. The column was packed with a predetermined amount of the adsorbent-sand mixture and exposed to varying operational parameters, including influent Cr(vi) concentrations (5, 10, 15, and 20 ppm), bed depths (5, 10, and 15 cm), and flow rates (2, 4, and 6 mL min^−1^). All experiments were conducted under ambient conditions, with the column continuously irradiated with UV light, as light-assisted adsorption can significantly influence Cr(vi) reduction and removal performance. Furthermore, the effluent samples were collected at regular intervals, and the Cr(vi) concentration was quantified using a UV-Vis spectrophotometer, following the same analytical procedure described for the batch experiments. The ratio of *C*_*t*_/*C*_o_ was plotted as a function of time to obtain breakthrough curves. The breakthrough time (*t*_b_) was defined as the point at which the effluent concentration reached approximately 5% of the influent concentration, while the exhaustion time (*t*_e_) corresponded to *C*_*t*_/*C*_o_ = 1. Additionally, the midpoint time (*t*_1/2_), representing *C*_*t*_/*C*_o_ = 0.5, was identified to understand the transition behaviour of the column (Ganji *et al.*, 2024).^21^ To interpret column performance and predict breakthrough parameters, dynamic kinetic models, such as the Adams–Bohart model (eqn (10)) and the Thomas model (eqn (11)), were applied).^24^ These models are instrumental for characterising adsorption dynamics, estimating kinetic constants, and understanding the efficiency and scalability of the fixed-bed column for Cr(vi) removal in continuous treatment systems.

## 3. Results and discussions

### 3.1 Characterisation of synthesised adsorbent

**Fig. 3:**
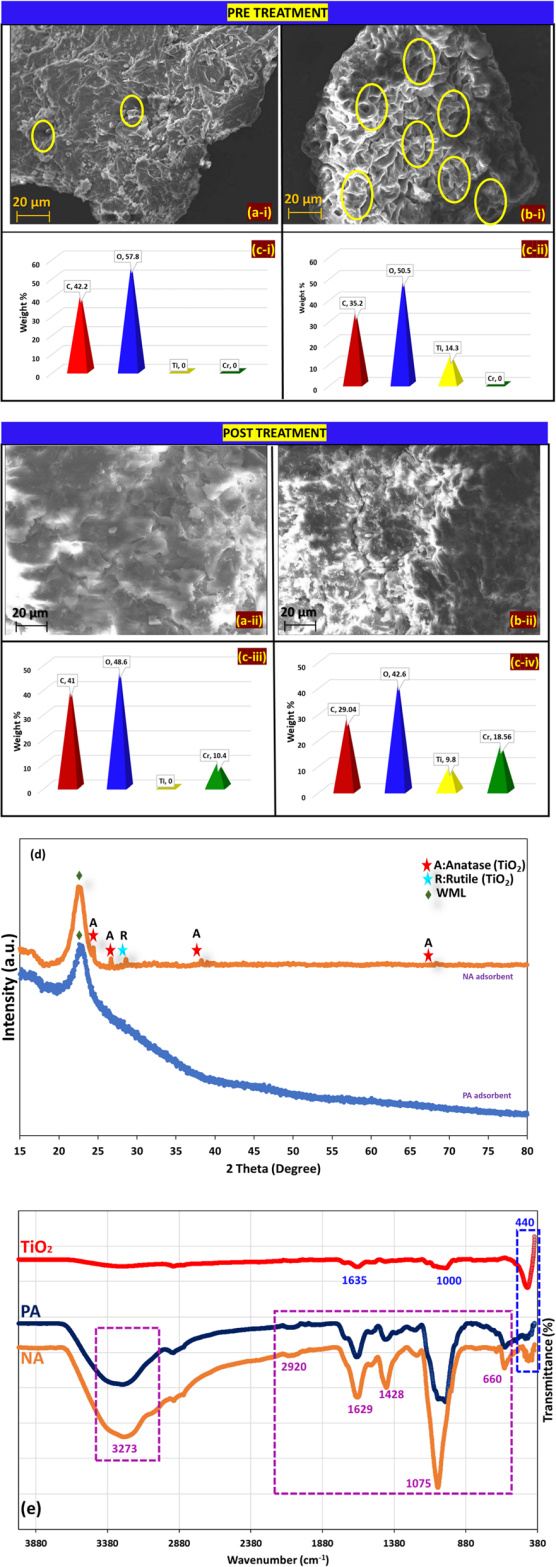
FESEM images of (a-i) PA and (b-i) NA adsorbent before treatment (a-ii) PA and (b-ii) NA adsorbent after treatment (c-i) EDAX plot of PA and (c-ii) NA adsorbent before treatment (c-iii) PA and (c-iv) NA adsorbent after treatment (d) XRD (e) FTIR spectra of TiO_2_, PA, and NA adsorbent.

The SEM, XRD, EDX, and FTIR analyses of the bio-adsorbent are illustrated in Fig. 3a–e. Fig. 3a collectively illustrates the detailed surface characteristics of the PA and NA adsorbents before and after Cr(vi) adsorption during batch experiments. These microscopic examinations provide critical insights into the morphological evolution of the materials and help elucidate the mechanisms governing their adsorption behaviour.^25^ Prior to Cr(vi) loading, the pristine PA adsorbent (Fig. 3a-i) exhibits a distinctly folded and irregular surface topology, characteristic of raw lignocellulosic biomass materials. The surface appears rough and non-uniform, suggesting the presence of naturally occurring micro-cavities, fibrils, and structural folds derived from the cellulose–hemicellulose matrix.^26^ Such intrinsic heterogeneity offers multiple binding sites that are favourable for physical adsorption processes; however, these features alone may not always support strong or specific interactions with metal ions without further surface modification.^25^ In contrast, the NA adsorbent (Fig. 3b-i) shows noticeably different surface features. The introduction of TiO_2_ NPs results in the development of clear, well-defined pores, along with enhanced textural complexity. The presence of these TiO_2_ NPs is evidenced by the increased porosity and rougher surface texture, which creates a higher surface-area-to-volume ratio.^1^ This structural enrichment is likely to improve the accessibility of active sites and enhance electrostatic and chemical interactions with Cr(vi) ions. Such modifications are crucial for promoting improved sorption performance and facilitating simultaneous photocatalytic activity (Aljeboree & Alkaim, 2024; B).^27^ Upon exposure to Cr(vi) during the batch adsorption process, PA and NA adsorbents undergo significant morphological transformations. The post-adsorption images (Fig. 3a-ii and b-ii) reveal that the previously visible pores and textural features become partially or completely obscured, resulting in smoother and more compact surface appearances. Ganji *et al.* (2024) observed that the porous structure of PHW was completely covered by Pb after adsorption.^21^ This change is attributed to the deposition or accumulation of Cr(vi) and its reduced species on the adsorbent surfaces. The disappearance or narrowing of the pores strongly suggests pore occupation, surface coverage, and possible chemical interactions between Cr species and the active functional groups present on the adsorbent matrices. In the case of the NA adsorbent, the reduction in visible porosity after adsorption may additionally indicate that TiO_2_ provides enhanced interactions, potentially through photocatalytic reduction, leading to stronger or more extensive metal deposition. These visual changes confirm that adsorbents exhibit effective uptake of Cr(vi), although the extent and nature of surface coverage differ depending on the material's structural properties. The changes observed in the morphology of the prepared adsorbent before and after treatment corroborate well with previous literature^19,28^

Energy-dispersive X-ray spectroscopy (EDX) analysis provides further confirmation of the adsorption behaviour and the compositional changes before and after Cr(vi) treatment. Fig. 3c(i–iv) shows the elemental profiles obtained from EDX, highlighting the distinct elemental composition of each adsorbent before and after treatment. Prior to adsorption, the NA adsorbent displays a clear signal for titanium, confirming the successful incorporation of TiO_2_ NPs into the biomass matrix. In contrast, the PA adsorbent contains only biomass-related elements, such as carbon and oxygen. After the adsorption process, Cr peaks appear in both PA and NA samples, providing direct evidence of chromium retention on their respective surfaces. The presence of Cr signals in the EDX spectra after treatment validates the successful adsorption of Cr(vi) and/or its reduced forms, particularly on the NA adsorbent, whereas TiO_2_-mediated photocatalytic reduction may further enhance metal accumulation. These compositional observations strongly corroborate the morphological transformations observed in the FESEM micrographs. Similar correlations between surface deposition and EDX-confirmed elemental composition have also been reported in the literature, including studies such as,^20,29^ which further reinforce the reliability of these findings.

X-ray diffraction (XRD) analysis was conducted to investigate the crystalline properties of both adsorbents, and the corresponding diffraction patterns are presented in Fig. 3d. The PA adsorbent exhibits a major broad diffraction peak at approximately 2*θ* = 21.50°, characteristic of amorphous cellulose structures.^26^ This broad peak reflects the disordered arrangement of cellulose chains, which is typical for untreated biomass materials. Such amorphous regions are known to provide functional groups, such as hydroxyl and carboxyl moieties, capable of interacting with metal ions during adsorption.^26,30^ In comparison, the NA adsorbent exhibits additional low-intensity crystalline peaks superimposed on the amorphous background. These peaks appear at 2*θ* values of 25.23°, 27.66°, 38.26°, and 70.41°. The peaks at 25.23°, 38.26°, and at higher angles (70.41°) correspond to the anatase phase of TiO_2_, in accordance with JCPDS Card No. 84-1285, while the peak at 38.26° match the rutile phase, consistent with JCPDS Card No. 46-1238.^31,32^ The predominance of the anatase phase is particularly significant, as anatase TiO_2_ is well known for its superior photocatalytic efficiency due to its high charge carrier mobility and slower electron–hole recombination rate.^33,34^ The coexistence of a minor rutile component may further enhance photocatalytic activity through the formation of anatase–rutile heterojunctions, which facilitate improved charge separation.^35^ The relatively low intensity of TiO_2_-related peaks suggests that the nanoparticles are embedded within the biomass matrix in moderate quantities, which may prevent particle agglomeration and maintain high dispersion across the surface of the NA adsorbent. Such dispersion is beneficial for maximising surface interactions with Cr(vi) ions.

The cataloguing of functional/active groups present in green synthesised TiO_2_ NPs, PA (pristine A-WML) and NA (A-WML/TiO_2_) nano adsorbent responsible for the adsorption of moieties was further analysed using FTIR, and results were illustrated in IR spectra (Fig. 3e). The characteristic absorption peak for TiO_2_ is usually observed at wavelengths below 800 cm^−1.^^23^ Obtained a peak at wavenumber 440 cm^−1^ corresponding to Ti–O–Ti stretching modes. A weak band observed around 1000 cm^−1^ indicates an O–Ti–O bond, whereas a crest around 1635 cm^−1^ corresponds to hydroxyl groups.^23^ The IR spectra of PA and NA adsorbents show almost similar peaks, with the NA adsorbent displaying one additional absorption peak. The possible additional peak, assigned to the O–Ti–O bond in TiO_2,_ appears at the previously assigned wavenumber and is found missing in PA adsorbent spectra. This further supports that TiO_2_ NPs were successfully embedded during the preparation of the NA adsorbent. Other common peaks observed in both PA and NA include a crest at 3200–3300 cm^−1^ is assigned to the vibration band –OH functional group of cellulose.^34^ Likewise, the small intensity peak observed at 2920 cm^−1^ signifies the C–H bond, whereas the absorption peaks appearing at 1629 and 1428 cm^−1^ are assigned to esters and the symmetric and asymmetric vibrations of –COO–. The crest at 1075 cm^−1^ signifies extending vibration CO–O–CO, and the peak around 660 represents the C=C bond.^31^ Identification of these groups from the FT-IR spectrum indicates the presence of a high abundance of carboxylic and hydroxyl groups, which actively participate as proton donors in binding cations.^26^

The combined SEM, EDX, XRD, and FTIR results provide a comprehensive understanding of the structural, compositional, and crystalline features of both adsorbents. The modifications introduced in the NA sample significantly alter its surface morphology, enhance porosity, and impart photocatalytic functionality through the incorporation of anatase-phase TiO_2_ NPs. These improvements directly contribute to enhanced adsorption and possible photoreduction of Cr(vi), making NA a more efficient adsorbent than PA. The distinct differences observed between the two materials before and after adsorption also illustrate the influence of surface preparation on metal uptake behaviour. Overall, the structural characterisation outcomes support the efficacy of the synthesised adsorbents and highlight the superior performance potential of the TiO_2_-modified NA variant. Such synergistic interactions between biomass-derived adsorption sites and photo-catalytically active TiO_2_ NPs underscore the relevance of green nanocomposite materials for sustainable heavy-metal remediation applications. Similar effects of NPs on enhancing the properties of bio-adsorbents have been reported in the literature.^36,37^

### 3.2 Surface area and pore size analysis

**Fig. 4:**
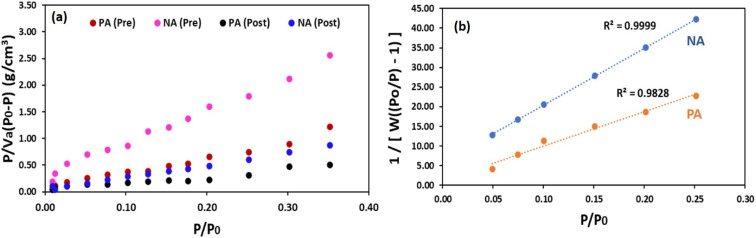
(a) BET plots of adsorbents before and after treatment (b) the BJH pore size distribution.

BET adsorption–desorption isotherm analyses were employed to determine the specific surface area, pore volume, and pore diameter of the PA and NA adsorbents before and after Cr(vi) uptake (Fig. 4a and b). The corresponding BET parameters are presented in Table 2. From the results, it was found that the NA adsorbent exhibits substantially higher surface area and porosity compared to the PA adsorbent. These findings align well with the FESEM observations (Fig. 3a-i and b-i), where the incorporation of TiO_2_ NPs resulted in more distinct and accessible pores. BET adsorption–desorption analysis revealed clear structural differences between the PA and NA adsorbents before and after Cr(vi) treatment. Before adsorption, the NA adsorbent exhibited a significantly higher surface area (98.25 m^2^ g^−1^), larger pore size (46.93 nm), and greater pore volume (0.256 cm^3^ g^−1^) than the PA adsorbent, which recorded 34.89 m^2^ g^−1^, 19.38 nm, and 0.082 cm^3^ g^−1^, respectively. A similar impact of NPs was also observed by ref. 7 where the surface area of chitosan (CS) increased from 25.42 to 63.67 m^2^ g^−1^ due to the inclusion of IONPs in the CS matrix. These differences confirm that NPs incorporation enhanced the structural porosity and prevented biomass aggregation, producing a more open, nanoparticle-stabilised matrix.^7^ Post-adsorption results showed a substantial decline in the surface area and pore dimensions of materials, with PA decreasing to 4.85 m^2^ g^−1^ and NA to 56.69 m^2^ g^−1^. This reduction reflects pore blockage and surface coverage by Cr(vi), confirming effective metal uptake. The overall trends strongly support the superior adsorption capability of NA and align with previously reported behaviour for nanoparticle-enhanced bio-adsorbents.^29,38^ Following Cr(vi) adsorption, adsorbents exhibited a marked reduction in surface area and pore diameter. This decline indicates that the available pore spaces were progressively filled or blocked by adsorbed Cr species, leading to the development of a surface coating on the adsorbent matrix. Such reductions in porosity and surface area provide strong evidence of effective metal uptake and are consistent with the morphological changes observed in the FESEM micrographs (Fig. 3a-ii and b-ii). BET and BJH analyses (Fig. 4a and b) revealed clear differences in the surface characteristics of PA and NA adsorbents before and after Cr(vi) treatment. NA exhibited a higher surface area and stronger linearity in BET fitting (*R*^2^ = 0.9999) compared to PA (*R*^2^ = 0.9828), indicating more efficient nitrogen adsorption and a well-defined mesoporous structure.^39^ After treatment, the adsorbents exhibited a reduced surface area and pore volume, confirming the occupation of pores by Cr(vi). NA retained relatively higher adsorption capacity post-treatment, highlighting its superior structural stability and enhanced suitability for Cr(vi) removal applications. The comparable trends in BET behaviour after metal adsorption have been reported by,^29,38^ further supporting the validity of these observations. Overall, the BET analysis confirms that TiO_2_ incorporation enhances the structural attributes of the NA adsorbent, resulting in improved accessibility of active sites and facilitating higher Cr(vi) adsorption performance.

**Table 2:** BET parameters for PA and NA adsorbents

Adsorbent	Surface area (m^2^ g^−1^)	Pore size (nm)	Pore volume (cm^3^ g^−1^)	*R* ^2^
PA (pre)	34.894	19.38	0.082	0.9680
NA (pre)	98.25	46.93	0.256	0.9990
PA (post)	4.85	8.56	0.009	—
NA (post)	56.69	21.85	0.015	—

### 3.3 Batch adsorption experiment

#### 3.3.1. Effect of contact time

**Fig. 5:**
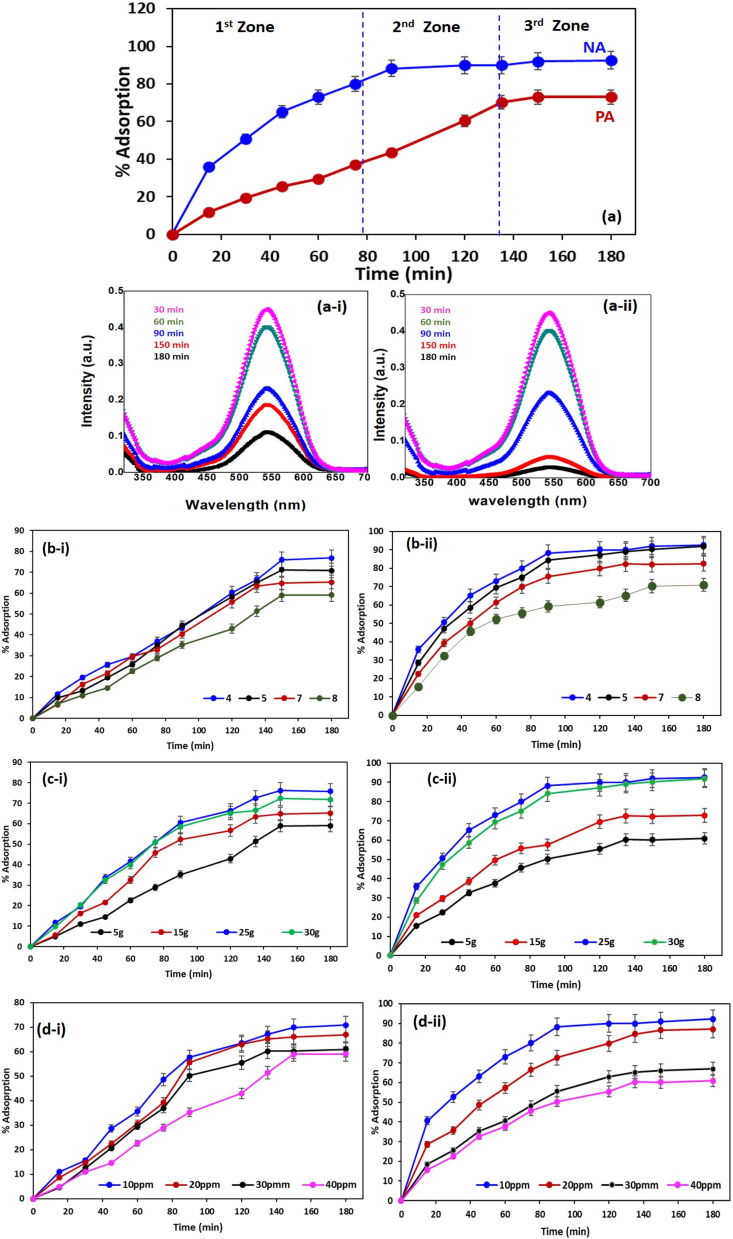
(a) Effect of the time variable on the percentage adsorption for PA and NA adsorbents (a-i) UV spectra of Cr in PA adsorbent and (a-ii) UV spectra of Cr in NA adsorbent at different time interval. Effect of (b) pH, (c) adsorbate dosage, and (d) adsorbent dosage on percentage adsorption for (i) PA and (ii) NA adsorbents.

The batch studies were conducted by adjusting key operational parameters, including contact time, pH, adsorbent dosage, and initial adsorbate concentration, to evaluate the adsorption performance of PA and NA. The adsorption kinetics and isotherm behaviour of Cr(vi) were examined in terms of percentage removal and adsorption capacity. The adsorption performance of NA and PA adsorbents toward Cr(vi) was evaluated as a function of contact time, revealing distinct adsorption behaviours across three kinetic zones (Fig. 5a). In the 1st zone (0–80 min), adsorbents exhibited rapid adsorption due to the abundance of available active sites.^14^ NA demonstrated a significantly faster uptake, reaching nearly 80% removal by 80 min, whereas PA showed a more gradual increase, achieving around 40–45% adsorption within the same interval. Furthermore, in the 2nd zone (80–140 min), the adsorption rate slowed as the active sites became progressively occupied.^14^ NA approached near-equilibrium levels (90–95%), reflecting efficient surface interactions and higher affinity for Cr(vi).^40^ PA continued to increase steadily, reaching approximately 70% removal by 140 min. In the 3rd zone (after 140 min), the adsorbents reached equilibrium, with minimal further increase in adsorption. NA stabilised above 95% removal, while PA plateaued around 75%. The consistently higher adsorption rate and final removal efficiency of NA throughout all zones indicate its superior surface characteristics and stronger interaction with Cr(vi). Overall, the kinetic trends highlight NA's enhanced adsorption capability compared to PA, particularly in terms of rapid uptake and higher equilibrium capacity. Similarly, different time zones were observed during the adsorption of pollutants such as organic and synthetic dyes, heavy metals *etc.*^41–43^Fig. 5a-i and ii represent UV spectra of Cr(vi) ion at different time interval in PA and NA adsorbent respectively. Spectra clearly depict decrease in absorbance value with increase in time due to adsorption of Cr ion, however decline in absorbance value was significantly higher when using NA adsorbent as already stated earlier attributed to combined effect of active components presents in bio-adsorbent along with presence of TiO_2_ NPs.

#### 3.3.2. Effect of pH

The pH of the Cr(vi) solution plays a crucial role in determining the surface charge of the adsorbents, thereby significantly influencing the adsorption efficiency of PA and NA. The influence of solution pH on Cr(vi) adsorption onto PA is clearly reflected in Fig. 5b-i, demonstrating that the surface protonation, speciation changes, and electrostatic interactions jointly determine the adsorption performance under varying acidic and alkaline conditions.^20,44^ As the pH increases from 4 to 8, a distinct downward trend in percentage adsorption is observed at all contact times. This pattern highlights the sensitivity of PA to pH-induced alterations in surface charge, which in turn controls the affinity between the negatively charged Cr(vi) species and the functional groups present on the adsorbent surface.^41^ At lower pH values (particularly pH 4 and 5), PA exhibits significantly higher adsorption compared to the performance recorded at neutral or alkaline pH. During the initial 20–40 min, the adsorption curve rises steadily, reflecting the availability of abundant active sites and strong electrostatic attraction between positively charged PA surfaces and predominant Cr(vi) anions such as HCrO_4_^−^ and Cr_2_O_7_.^2–40^ These conditions favour rapid uptake, enabling the adsorption percentage to surpass 40% within the first hour at pH 4. As pH increases to 7 and 8, the upward trajectory during the early stages becomes more gradual, indicating a decline in the driving force for adsorption. As time proceeds into the mid-phase (60–120 min), the gap between acidic and neutral/alkaline curves becomes more pronounced. At pH 4, PA continues to accumulate Cr(vi) efficiently, ultimately approaching near-plateau levels of around 70–75% after 120 min. In contrast, at pH 7 and 8, the adsorption remains considerably lower, with values typically not exceeding 40–45% even at extended contact times. This behaviour is strongly linked to the proximity of PA's point of zero charge (pH_PZC_), at which the surface transitions from positively charged to negatively charged.^26^ Above the pH_PZC_, PA develops negative surface sites, which electrostatically repel the predominant CrO_4_^2−^ and Cr_2_O_7_^2−^ ions, resulting in a substantial reduction in uptake. The final phase (beyond 120 min) shows the adsorption curves approaching equilibrium. Even at this stage, the separation between the pH conditions remains substantial. At pH 4, PA achieves its maximum removal efficiency, between 75–80%, whereas at pH 8 the adsorption stabilises at only 20–25%. This substantial decrease demonstrates that alkaline conditions are strongly unfavourable for Cr(vi) uptake. Under higher pH, not only does electrostatic repulsion dominate, but competition from hydroxide ions also becomes more significant, further reducing the available adsorption sites for Cr(vi) binding. Overall, Fig. 5b-i reveals that the adsorption efficiency of PA is highly pH-dependent, with acidic conditions strongly favouring Cr(vi) removal due to enhanced protonation and strong electrostatic attraction.^26^ As the pH shifts towards neutral and alkaline ranges, the adsorption efficiency steadily declines, indicating the transition from electrostatic attraction to repulsion. These results emphasise that PA performs optimally under acidic environments and loses effectiveness when the surface charge becomes negative at elevated pH levels.^26^

The influence of solution pH on Cr(vi) adsorption using NA is illustrated in Fig. 5(b-ii), showing a more pronounced response compared to PA due to the enhanced surface charge characteristics imparted by the TiO_2_ NPs incorporated into the adsorbent. Across all pH conditions tested (pH 4, 5, 7, and 8), NA consistently exhibits higher adsorption efficiencies than PA, reflecting the improved electrostatic and surface interaction mechanisms provided by the composite structure. At pH 4, NA exhibits exceptionally high adsorption, with uptake exceeding 60% within the first 40 min. This rapid increase is attributed to the strong positive charge present on the NA surface under acidic conditions, particularly due to the formation of protonated Ti–OH_2_^+^ groups.^39,40^ These positively charged sites dramatically enhance the electrostatic attraction between the surface and the negatively charged Cr(vi) species. As the reaction proceeds into the mid-contact period (40–80 min), the adsorption rises sharply, reaching values above 80%. When the pH is increased to 5, the adsorption capacity remains high but slightly reduced compared to pH 4. The adsorption curves maintain a similar rising pattern, albeit with a more moderate slope, indicating that the surface charge density remains favourable but has begun to decline as the pH approaches the pH_PZC_. Nevertheless, NA continues to reach 75–85% adsorption after an extended contact time, indicating that its modified surface provides a high affinity even under mildly acidic conditions. Furthermore, at pH 7 and 8, the behaviour of NA contrasts clearly with the acidic range. Although the initial adsorption rate is still relatively strong due to the presence of residual surface functional groups capable of interacting with Cr(vi), the overall performance is significantly reduced. At pH 7, the adsorption increases steadily to around 60–70% after 180 min. However, at pH 8, the maximum adsorption does not exceed 40–45%. This decline reflects the reduction in positive charge and the onset of electrostatic repulsion between the NA surface and Cr(vi) anions under alkaline conditions.^10^ The enhanced performance of NA across the entire pH range compared to PA can be attributed to the amphoteric behaviour of TiO_2_ nanoparticles.^31^ These nanoparticles introduce additional active sites capable of protonation under acidic conditions, thereby significantly increasing the surface's capacity to attract and bind Cr(vi).^33^ Even as the pH increases, NA retains more favourable surface characteristics than PA, resulting in better adsorption under neutral and slightly alkaline conditions. Nonetheless, the progressively increasing concentration of OH^−^ ions at higher pH competes with Cr(vi) for adsorption sites and shifts the surface charge potential, ultimately reducing the overall uptake. Towards the equilibrium phase (after 120 min), NA achieves nearly 90–95% adsorption at pH levels of 4 and 5, while maintaining moderate efficiency at pH levels of 7 and 8.^26,30,44^ These results underscore the superior pH tolerance of NA compared to PA, highlighting the beneficial role of TiO_2_ NPs, particularly in acidic environments. Table 3 shows the possible reaction that happened during the adsorption.

Charge density over the adsorbent surface and Cr(vi) ion at different pH levels and their effect^26,30,44^

**Table:** 

	pH range	Probable forms	Equations
Cr(vi)	1 to 7	Cr_2_O_7_^2−^, HCrO_4_^−^	H_2_CrO_4_ + H_2_O → H_3_O^+^ + HCrO_4_^−^
			HCrO_4_^−^ + H_2_O → H_3_O^+^ + HCrO_4_^2−^
	>7	CrO_4_^2−^	2HCrO_4_^−^ → H_2_O + Cr_2_O_7_^2−^
TiO_2_ NPs	pH < PZC (∼4.3)	Ti–OH_2_^+^	TiO_2_ + H^+^ ⇌ Ti–OH_2_^+^
	pH > PZC (∼4.3)	Ti–O^−^	TiO_2_ + OH^−^ ⇌ Ti–O^−^ + H_2_O
A-WML	pH < PZC (∼6.5)	+ve charge	—
	pH > PZC (∼6.5)	−ve charge	—

**Table:** 

	Overall Effect						
	PA adsorbent			NA adsorbent			
	Cr(vi) ion charge	A-WML surface charge	Net effect	Cr(vi) ion charge	A-WML surface charge	TiO_2_ surface charge	Net effect
Acidic pH (<5)	−ve	+ve	Electrostatic Interaction	−ve	+ve	+ve	More intense electrostatic interaction
Basic pH (>7)	−ve	−ve	Repulsive force	−ve	−ve	−ve	Repulsive force

#### 3.3.3. Effect of adsorbent dosage on Cr adsorption

Fig. 5c-i displays the influence of varying PA dosages (5–30 g) per liter on Cr(vi) adsorption. The results demonstrate a clear positive relationship between adsorbent dosage and removal efficiency, though with noticeable differences in adsorption kinetics and equilibrium values across dosage levels. At lower dosages, such as 5 and 10 g, the initial adsorption rates are relatively slow, with early-stage removal remaining below 20% for the first 30 min. This is attributed to the limited number of available adsorption sites per unit volume, which restricts the interaction between Cr(vi) ions and the PA surface.^20,45^ As the dosage increases to 15, 20, 25, and 30 g, the initial adsorption rate improves significantly. For instance, adsorption exceeds 40% within the first 40 min at a concentration of 30 g, reflecting the increased abundance of active sites and enhanced surface accessibility.^43^ Higher dosages introduce more binding regions, enabling faster uptake of Cr(vi) even at early contact times.^20,24,25^ During the mid-phase (40–120 min), the effect of dosage becomes more pronounced. The 5 g dosage shows a gradual increase, reaching approximately 35–40% by 120 min. In contrast, higher dosages such as 25 and 30 g reach 60–70% in the same duration. However, the curves also reveal that beyond a certain threshold, the incremental benefit of increased dosage begins to taper off. This is attributed to particle agglomeration, reduced effective surface area due to overlapping sites, and intraparticle diffusion limitations.^43^ Furthermore, in the equilibrium phase (120–180 min), the adsorption performance stabilises across all dosages. At 30 g, PA achieves a maximum removal of nearly 75%, while the lowest dosage (5 g) stabilises around 45%. This clearly confirms that increasing the adsorbent dosage enhances Cr(vi) removal; however, the relationship is nonlinear, with diminishing returns at higher dosages.^46^ A similar effect of increasing adsorbent dosage in enhancing the adsorption performance of sewage sludge-derived adsorbent for dye was reported by Wang *et al.* 2024.^46^

Fig. 5(c-ii) shows the influence of varying NA dosages (5–30 g) per liter on Cr(vi) adsorption. It was found that across all dosages, NA consistently outperforms PA due to its higher surface area, improved charge characteristics, and the presence of TiO_2_ NPs. At lower dosages (5 and 10 g), NA exhibits faster initial kinetics compared to PA, with adsorption exceeding 25–30% within the first 20 minutes. Further, increasing the dosage (30 g) dramatically enhances performance; the adsorption exceeds 50% within the first 30 min, highlighting the superior binding capabilities of the nano-adsorbent. In the mid-contact stage (40–120 min), higher dosages show significantly enhanced removal efficiencies. For example, at 25 and 30 g, NA achieves 75–85% adsorption by 120 min, whereas lower dosages reach only 50–60%. The improved performance is attributed to increased active site availability, stronger electrostatic attraction, and the facilitated interaction provided by TiO_2_ NPs.^47^ At equilibrium (120–180 min), NA reaches maximum removal efficiencies of 90% at 30 g L^−1^ and 80–85% at 25 g. Even the lowest dosage, 5 g, stabilises around 60%, outperforming PA at similar dosages. These results confirm the superior adsorption characteristics imparted by the nanocomposite structure.

#### 3.3.4. Effect of Cr(vi) concentration on performance of PA and NA

Fig. 5d-i illustrates the adsorption behaviour of PA at various initial Cr(vi) concentrations (10–40 ppm). At lower concentrations (10 and 20 ppm), PA exhibits relatively rapid adsorption kinetics, achieving nearly 40% removal within the first 40 min. As concentration increases to 30 and 40 ppm, the initial adsorption rate declines, as indicated by slower initial slopes and lower uptake percentages. During the intermediate contact period (40–120 min), the effect of concentration becomes more pronounced, with adsorption progressing steadily toward 60–70% at a concentration of 10 ppm. Meanwhile, at 40 ppm, despite the driving force being higher, the removal percentage remains substantially lower (20–30%). This is due to the limited availability of adsorption sites and early surface saturation.^31,40^ At equilibrium (120–180 min), PA reaches 75% removal at 10 ppm, 65% at 20 ppm, and significantly lower values (35–40%) at higher concentrations (30–40 ppm). This demonstrates that increasing Cr(vi) concentration results in decreased percentage removal due to saturation constraints and competitive site occupation.^26^Fig. 5(d-ii) shows the effect of varying Cr(vi) concentrations on the adsorption performance of NA. Results confirmed that NA consistently outperforms PA across all concentrations, maintaining high affinity and rapid uptake due to its enhanced surface chemistry. At lower Cr(vi) concentrations (10 and 20 ppm), NA rapidly adsorbs Cr(vi), achieving more than 50% uptake within the first 30 min and progressing to 80–90% by 120 min. At higher concentrations (30 and 40 ppm), the initial uptake remains relatively high due to the strong electrostatic attraction, but it slows over time as the available sites become occupied. In the final equilibrium phase (120–180 min), NA achieves 90–95% removal at 10 ppm, 85–90% at 20 ppm, and 60–70% even at 40 ppm, significantly outperforming PA at similar concentrations. This highlights NA's stronger adsorption capacity and enhanced tolerance to concentration increases.^47^

##### 3.3.5 Adsorption kinetics

###### 3.3.5.1 Kinetics study (linear form)

**Fig. 6:**
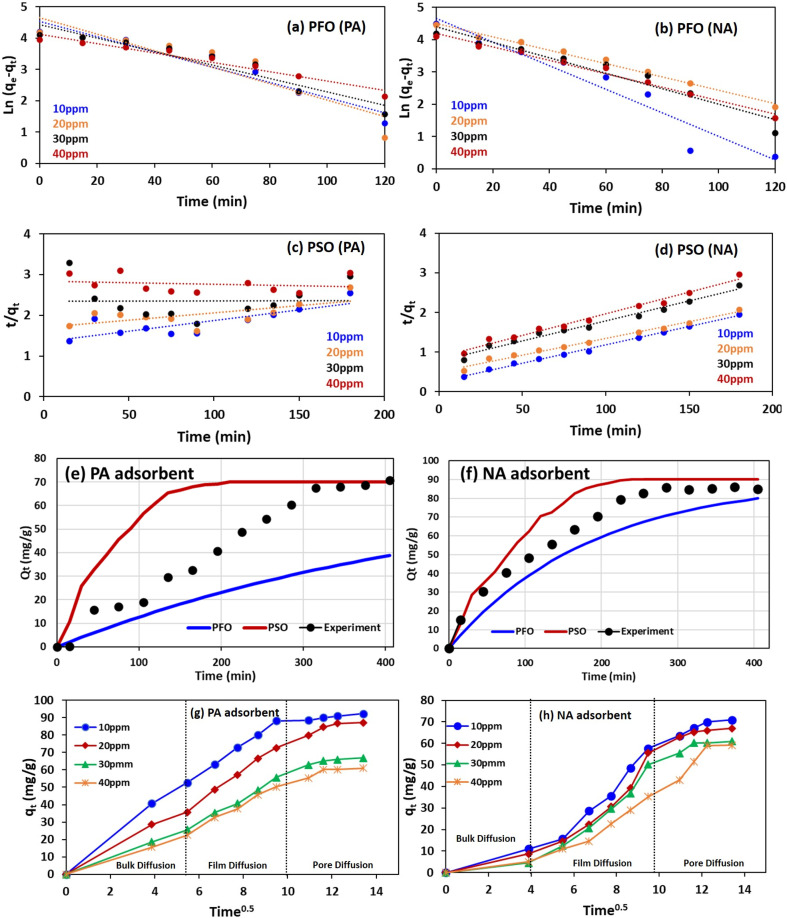
Kinetics curves (Linear) at different concentrations for (a) PFO for PA, (b) PFO for NA, (c) PSO for PA, (d) PFO for NA adsorbent. Kinetics curves (non-linear) for (e) PA and (f) NA adsorbent. Non-linear IPD kinetics curve for (g) PA and (h) NA adsorbent.

This section highlights the significance of adsorption kinetic models, namely, the pseudo-first-order and pseudo-second-order models, in determining the rate-controlling steps governing the interaction between the adsorbent and adsorbate. Pseudo-first-order (PFO) states that the adsorption rate is solely dependent on the concentration of the adsorbate (liquid phase).^42^ On the other hand, in the pseudo-second-order (PSO) model, the adsorption rate accounts for adsorbate and adsorbent concentrations.^20,48^ The kinetics were investigated for the present system, and the results are illustrated in Fig. 6. The experiment was performed at different Cr concentrations, varying from 10 to 50 ppm, and the findings indicate that Cr ion sorption onto PA and NA exhibits better fitting with PSO kinetics and is more comprehensive, as indicated by the higher regression coefficient value, *i.e.*, *R*_1st order_^2^ < *R*_2nd order_^2^. It simply means that the adsorption of Cr ions is primarily governed by chemisorption (the rate-limiting step).^41^ The evaluated constant parameters for both models are summarised in Table 4.

**Table 4:** Evaluated kinetic model (linear) parameters

Adsorbent	Cr conc. (ppm)	Pseudo first order (PFO)			Pseudo-second order (PSO)		
		*q*_e_ (mg g^−1^)	*K*_1_	*R* ^2^	*q*_e_ (mg g^−1^)	*K*_2_	*R* ^2^
PA	10	5.30	0.0244	0.931	7.54	0.080	0.910
	20	22.63	0.0263	0.849	22.35	0.165	0.879
	30	25.96	0.0214	0.915	26.85	0.192	0.895
	40	32.54	0.0149	0.950	35.55	0.185	0.890
NA	10	9.87	0.0365	0.951	9.11	0.262	0.991
	20	18.97	0.0207	0.958	25.63	0.364	0.998
	30	28.27	0.0239	0.963	38.56	0.565	0.983
	40	39.54	0.0194	0.953	45.96	0.791	0.993

Key observation from Tables 4 and 5 is mentioned below;

**Table 5:** Evaluated kinetic model (non-linear) parameters

Adsorbent	Pseudo first order (PFO)			Pseudo-second order (PSO)		
	*K*_1_	*R* ^2^	*χ* ^2^	*K*_2_	*R* ^2^	*χ* ^2^
PA	0.023	0.851	28.12	0.081	0.891	17.82
NA	0.039	0.905	25.13	0.298	0.961	11.85

• The rate constant follows the order (PSO > PFO), which signifies that the PSO model provides a more accurate representation of the experimental data.

• The PSO rate constant (*K*_2_) for the NA adsorbent is higher than that for PA, indicating faster Cr(vi) uptake, likely due to the enhanced reactivity and intercalation effects of nanoparticles in NA.^18,40^

• Another interpretation from the table is that the coefficient of correlation for the NA adsorbent was >0.95 for all different concentrations, and consistently higher than the PA adsorbent. This further ensures the PSO model is more suitable for the NA adsorbent compared to PA.

In conclusion, these results suggest that the mechanism behind Cr(vi) adsorption may occur through a combination of the electrostatic, ion exchange, and reduction processes.^49^

###### 3.3.5.2 Kinetics study (non-linear form)

A kinetic study in non-linear form provided a more realistic solution to the existing system. Evaluated values from the non-linear study indicate that the PSO model provides a better curve fitting (Fig. 6e and f), as the non-linear regression coefficient value (Table 5) is higher for PSO compared to the PFO model for the existing system. Also, the observed Chi-square test (*χ*^2^) values obtained for the PSO model are lower than the PFO model, further confirming that the PSO model is suitable kinetics of this system.^49^

Although the PSO model provided a better fit for the present system, it still does not accurately define the diffusion mechanism. To better understand this, the interparticle diffusion (IPD) kinetic model was studied, and its parameters (eqn (12)) are evaluated and compared with experimental data.

12 *Q*_*t*_ = *K*_IPD_*t*^1/2^ + *C*

where *K*_IPD_ is the interparticle diffusion factor (mg g^−1^ min^−0.5^).

Graph shown in Fig. 6g and h illustrate that IPD occurs in three phases. Firstly, Cr is diffused from the bulk solution to the interface, followed by infusion into capillaries, and lastly diffusion *via* micro pores to attain the highest adsorption capacity. Since a line passing through the origin is observed in both PA and NA adsorbents, it indicates that the rate-limiting step is purely IPD. It was also observed that with increasing concentration of Cr, the thickness of the boundary layer is remarkably increased, thus increasing the pathway period, thereby reducing the adsorption capacity at a given time.^49^

##### 3.3.6 Adsorption Isotherm

**Fig. 7:**
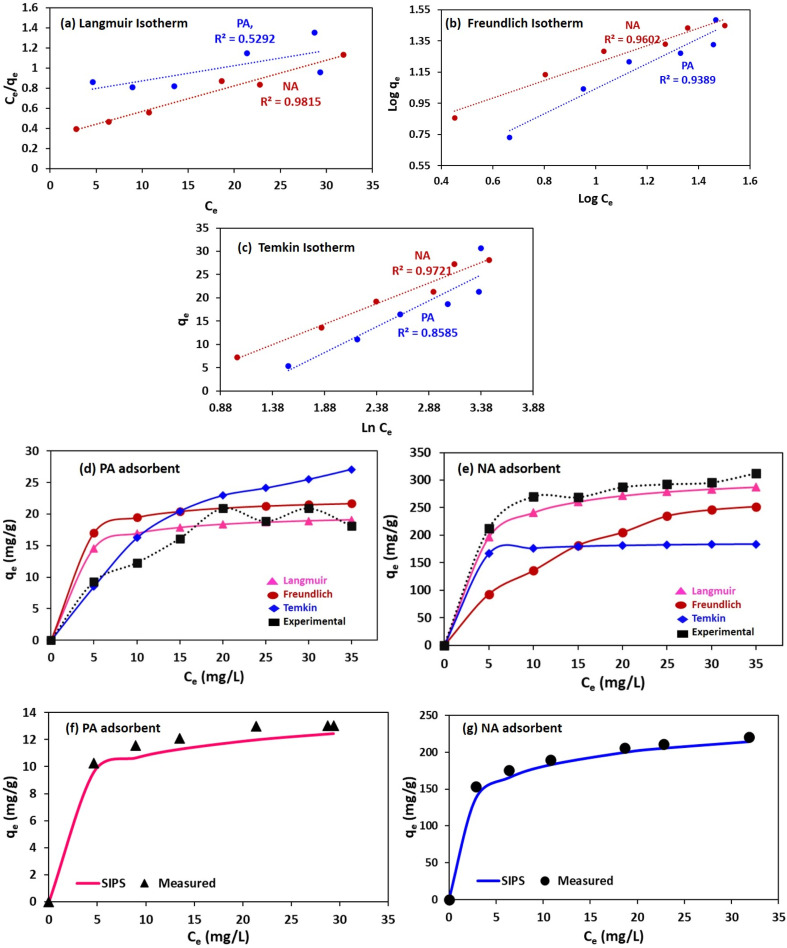
Linear model (a) Langmuir model, (b) Freundlich model, (c) Temkin model of Cr(vi) onto PA and NA adsorbent. Non-linear isotherm model (d) PA and (e) NA adsorbent. SIPS (non-linear) isotherm model for (f) PA and (g) NA adsorbent.

The isotherm studies provide valuable insight related to the distribution of Cr ions between the solid phase and the solution at a certain temperature in equilibrium, based on *q*_e_ (mg g^−1^) and *C*_o_ (mg L^−1^) values. In this study, equilibrium data for Cr(vi) adsorption were assessed using the Langmuir, Freundlich, and Temkin isotherms.^6,41^ The corresponding results of the Linear form of isotherms are illustrated in Fig. 7a–c, and the fitted model parameters are summarised in Table 6. The graph indicates that the NA adsorbent provides a better isotherm fit compared to the PA adsorbents. Among the three models, the Langmuir isotherm (in linear form) for NA provided the highest correlation coefficient (*R*^2^ = 0.9815) for the removal of Cr ions, thereby suggesting monolayer adsorption on a homogeneous surface.^41^ The adsorption of Cr ion on the NA adsorbent can be strongly justified by the Langmuir constant (*K*_L_) value ranging from 0.79 to 6.49 L mg^−1^. The high value of *q*_m_ model for Cr, predicted by the Langmuir, further justifies and demonstrates the strong potential of the NA adsorbent in removing Cr ions from the aqueous solution compared to the PA adsorbent.^48,49^ The other model, the Freundlich isotherm, assesses how well the adsorbents removed the Cr ion from solution, with its constant reflecting binding affinity. The linear fit values of this model consider the adsorption process as a heterogeneous phenomenon; also, the evaluated binding affinity for Cr was higher for the NA adsorbent compared to PA. The other information drawn is that the adsorbents possess a strong affinity for Cr(vi) ions since 1/*n* value < 1 (Table 6), further confirming that the adsorption process is favorable.^12,49^ The Temkin isotherm model describes the indirect effect of adsorbent–adsorbate interactions on the adsorption process. Positive value of *B*_T_ > 0 signifies that the adsorption is exothermic, and a high value of *K*_T_ for the NA adsorbent implies a stronger interaction between Cr and the NA adsorbate surface compared to the PA adsorbent. Overall, results conclude that Cr adsorption on PA and NA is favourable (*R*_L_ < 1), predominantly follows the Langmuir model, involves monolayer coverage, and is removed *via* chemisorption.^12,41^

Evaluated parameters for different linear and non-linear isotherms

**Table:** 

Adsorbents	Linear form									
	Langmuir isotherm				Freundlich isotherm			Temkin isotherm		
	*K*_L_	*q*_m_	*R* ^2^	*R*_L_	*K*_F_	1/*n*	*R* ^2^	*K*_T_	*B*_T_	*R* ^2^
PA	0.89	22.58	0.5292	0.9	1.55	0.895	0.9389	0.95	23.65	0.8585
NA	1.92	162.21	0.9815	0.89	11.95	0.925	0.9602	1.12	35.82	0.9721

**Table:** 

	Non-linear form								
	*K*_L_	*R* ^2^	*χ* ^2^	*K*_F_	*R* ^2^	*χ* ^2^	*K*_T_	*R* ^2^	*χ* ^2^
PA	0.92	0.90	12.85	1.71	0.86	15.12	0.98	0.87	17.82
NA	2.05	0.99	9.12	13.62	0.89	11.96	1.21	0.909	5.85

**Table:** 

	SIPS model			
	*K*_s_	*m*	*R* ^2^	*χ* ^2^
PA	0.251	0.34	0.8792	4.56
NA	0.326	0.51	0.9240	3.21

Herein, a non-linear isotherm study was also carried out to provide a better fit to the models. The obtained results were plotted as shown in (Fig. 7d and e), and the calculated parameters were tabulated in Table 6. The highest non-linear regression coefficient (*R*^2^ = 0.99), indicating a good fit with the Langmuir model compared to other models. The observed lowest value of the Chi-square test (*χ*^2^ = 9.12) for the Langmuir model further confirms that this model is suitable for the current study.

Since non-linear Langmuir and Freundlich reflect better correlation with experimental data, combined Langmuir and Freundlich (SIPS) model was also studied further to correlate with given data. The SIPS equation is a multidimensional equation (eqn (13)) that predict behaviour of both above mentioned model in heterogeneous system.13
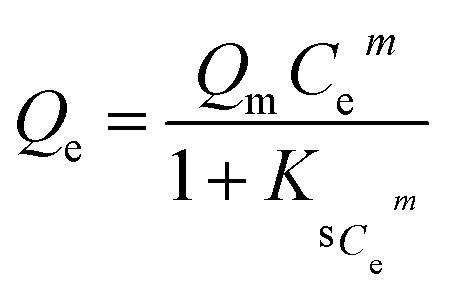
where *Q*_e_ and *Q*_m_ are equilibrium and maximum adsorption capacity, *C*_e_ (mg L^−1^) is equilibrium concentration, *K*_s_ (L mg^−1^)^*m*^ is SIP isotherm constant and *m* is the empirical constant. However, based on highest *R*^2^ value obtained in SIPS isotherm model (Table 6) among other non-linear model, proves SIPS model to be best fitted model with equilibrium data.

### 3.4 Desorption studies

**Fig. 8:**
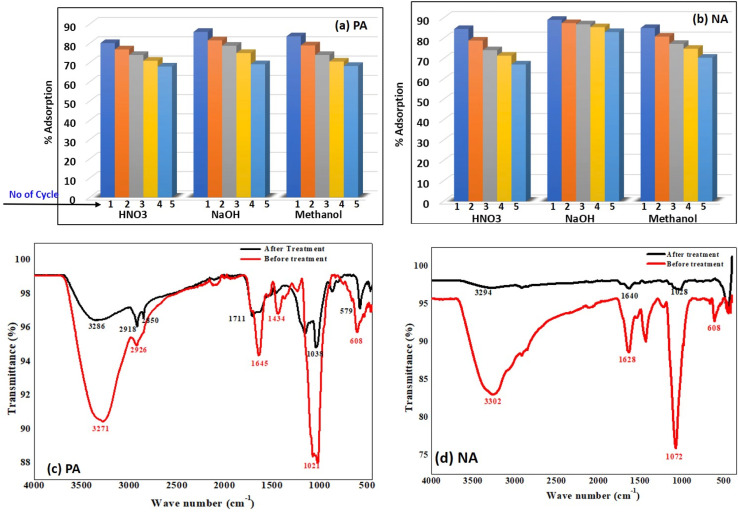
Reusability plot of (a) PA and (b) NA adsorbents under different solutions. FTIR plot of (c) PA and (d) NA adsorbents before and after treatment.

The regeneration performance of PA and NA adsorbents was evaluated over five consecutive adsorption–desorption cycles using three different regenerating agents: HNO_3_, NaOH, and methanol, to understand their stability, reusability, and sustained efficiency in Cr(vi) removal. Fig. 8a and b demonstrate that the adsorbents retained high adsorption efficiency across multiple cycles. Although a gradual decline was observed with repeated use, a common phenomenon in regeneration studies due to the progressive saturation and minor structural changes that occur in the adsorbent matrix.^50^ For PA, the first cycle consistently exhibited the highest adsorption efficiency regardless of the regenerant used. Furthermore, HNO_3_ was applied, and PA exhibited an initial adsorption performance of approximately 82–85%, indicating effective desorption and restoration of active surface sites. However, the slight decreases appeared with each subsequent cycle, reaching approximately 72–74% by the fifth cycle. This progressive reduction suggests partial occupation of active binding sites by strongly retained chromium species or mild structural fatigue caused by repeated acid exposure.^50,51^ The NaOH-regenerated PA exhibited similar trends but demonstrated marginally superior retention of adsorption capacity compared to HNO_3_. The first cycle exhibited the highest adsorption efficiency (approximately 86–88%), followed by a steady yet slightly lower decline across subsequent cycles. By the fifth cycle, the adsorption performance remained around 74–76%, indicating that alkaline regeneration may better preserve the integrity of PA's functional groups, particularly those susceptible to acid-induced degradation. Methanol regeneration exhibited comparable trends to PA, although its initial adsorption percentage was slightly lower than that of HNO_3_ and NaOH. The first cycle demonstrated adsorption efficiencies ranging from 83% to 85%, with a moderate decrease observed across subsequent cycles. By the 5th cycle, adsorption stabilised around 73–75%. Methanol, functioning primarily as an organic solvent, may have selectively desorbed certain chromium complexes but did not fully restore all active adsorption sites as effectively as the acid or alkali regenerants.^52^ For NA, the results revealed a similar pattern but with slightly better retention of adsorption capacity across all regeneration cycles compared to PA. Under HNO_3_ regeneration, NA displayed an initial adsorption efficiency of approximately 88–90%, indicating a strong affinity between NA's functional surface and Cr(vi). Despite a gradual decline, NA maintained adsorption values near 75–77% by the fifth cycle, demonstrating good structural resilience even under acidic desorption conditions. Furthermore, NA showed its best regenerative performance under NaOH treatment. The adsorption efficiency remained exceptionally high during the first cycle (close to 90%), with minimal deterioration across cycles. Even by the fifth cycle, the adsorption percentage remained relatively high (78–80%), confirming that the alkaline medium effectively restored active binding sites with minimal structural alteration.^51^ Methanol regeneration for NA also showed robust performance with an initial efficiency of around 88–89%, tapering slightly to 74–76% by the fifth cycle. Although methanol was not as effective as NaOH, its performance was comparable to that under HNO_3_, showing that NA can maintain stability across different regenerants. Overall, the results confirm that PA and NA possess strong reusability potential, with NA demonstrating slightly superior regeneration stability. Additionally, NaOH proved to be the most effective regenerant for maintaining adsorption performance in both materials.

To determine whether the adsorbent material undergoes any structural changes before and after treatment, the material was characterised using FTIR. FTIR spectra of PA and NA adsorbents before and after treatment are shown in Fig. 8c and d. It is quite evident from the figure that after treatment, the peak intensity declines at 3271 cm^−1^, 1645 cm^−1^, and 1021 cm^−1^ in the PA adsorbent, while a decrease in peak intensity is observed at 3302 cm^−1^, 1628 cm^−1^, and 1072 cm^−1^, which signifies the adsorption of heavy metal on the active components. Similarly, peak shift is also observed from 3271 to 3286 cm^−1^ (–OH group), 1645 to 1711 cm^−1^, 1021 to 1038 cm^−1^ (esters and –COO), in PA adsorbent, while peak shift is observed from 3302 to 3294 cm^−1^ (–OH group), 1628 to 1640 cm^−1^ (esters and –COO), 1072 to 1028 cm^−1^ (CO–O–CO group) again due to participation of active compounds in binding Cr ion on its surface.^31^ These structural changes further confirm that the pollutant is effectively bound onto the surface of the adsorbent material.

### 3.5 Photocatalytic experiments

**Fig. 9:**
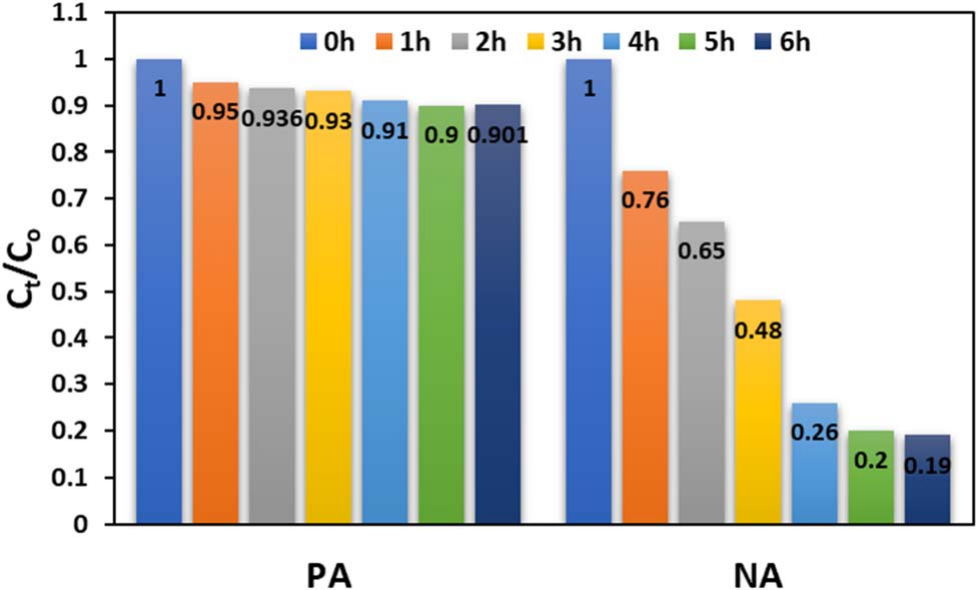
Time-dependent concentration chart for Cr(vi) using PA and NA nano-adsorbent.

The temporal variation in the normalised concentration ratio (*C*_*t*_/*C*_o_) provides a clear insight into the adsorption kinetics and comparative adsorption efficiency of the PA and NA adsorbents over a 6 h period. The result of the photocatalytic activity of the adsorbents was depicted in Fig. 9. The plotted data reveal two distinct kinetic behaviours: PA shows a slow and gradual decline in *C*_*t*_/*C*_o_, whereas NA demonstrates a rapid and significant reduction over time. This contrast highlights the major functional and structural differences between the two adsorbents in their ability to remove Cr(vi) from solution. For PA, the *C*_*t*_/*C*_o_ ratio at the initial time (0 h) starts at 1.0, reflecting the untreated initial concentration. After 1 h, PA shows only a marginal reduction to 0.95, revealing its slow adsorption initiation phase. At 2 h, *C*_*t*_/*C*_o_ further decreases slightly to 0.93, and by 3 h, it reaches 0.91. These small changes indicate that PA possesses moderate adsorption activity that progresses gradually, likely governed by slower surface interaction processes and controlled diffusion within its pores.^22^ Furthermore, between 3 and 6 hours, the trend stabilises, with *C*_*t*_/*C*_o_ values fluctuating between 0.90 and 0.91. This near-steady behaviour suggests that PA approaches equilibrium early, with limited additional Cr(vi) uptake beyond the initial few hours. The relatively minor drop in *C*_*t*_/*C*_o_ from 1.0 at 0 h to approximately 0.90 at 6 h implies that PA has a lower affinity and fewer effective active sites available for rapid Cr(vi) adsorption.^36^

In contrast, NA exhibits a markedly different adsorption profile, characterised by a rapid and strong reduction in *C*_*t*_/*C*_o_ values within the same time frame. At 0 h, the concentration returns to baseline (1.0), but by 1 h, it drops sharply to 0.76, indicating a swift adsorption response. The *C*_*t*_/*C*_o_ further decreases to 0.65 by 2 h, demonstrating that NA quickly captures a substantial proportion of Cr(vi) from the solution. The downward trend continues more aggressively than in PA, with the *C*_*t*_/*C*_o_ reaching 0.48 at 3 h. This steep decline clearly shows NA's higher density of active functional groups and superior accessibility of adsorption sites. The value reaches 0.26 by 4 h, marking an advanced stage of pollutant removal. The most significant drop is observed between 4 and 6 hours, where *C*_*t*_/*C*_o_ reduces further to 0.20 and then to 0.19. This near-complete reduction indicates that NA achieves almost full adsorption equilibrium within the 6 h duration, significantly outperforming PA. Overall, the comparison demonstrates that NA is a much more effective and kinetically favourable adsorbent for Cr(vi) removal than PA. The PA shows modest adsorption with minimal reduction over time. NA's rapid and consistent decline in *C*_*t*_/*C*_o_ highlights its strong surface affinity, higher porosity, and more reactive functional groups. The substantial reduction in NA's *C*_*t*_/*C*_o_ from 1.00 to 0.19 in 6 h confirms its superior capacity and faster adsorption kinetics. This distinction highlights NA's suitability for applications that require rapid and efficient contaminant removal, whereas PA may be more suitable for slower, longer-contact systems.

### 3.6 Column fixed-bed adsorption experiments

Adsorption column efficiency is a function of several parameters, with column bed height, feed flow rate, and adsorbate concentration being the most significant.^53^ Bed height directly affects removal efficiency and breakthrough period. Increasing bed height provides more active adsorption sites, allowing for a longer contact time between the adsorbate and adsorbent, thereby promoting the binding of metals to available active sites and enhancing column removal efficiency, as well as increasing the breakthrough time.^54^ However, an optimum bed height is desirable for operational feasibility, as an excessive bed height produces a drawback in terms of increased pressure drop. Similarly, the feed flow rate is another controlling factor that influences the residence time of the influent solution in the column. Low flow rates generally increase contact times, allowing for better diffusion of metal into the adsorbent matrix, which ultimately improves removal efficiency and delays the breakthrough period. In contrast, higher flow rates cause early bed saturation due to insufficient interaction time and poorer performance.^12^ Influent concentration is another crucial parameter in defining the column dynamics. High metal concentration increases the driving force for mass transfer, enabling the rapid uptake of contaminants at the expense of early bed saturation due to reduced residence time, resulting in quicker breakthrough.^21^

**Fig. 10:**
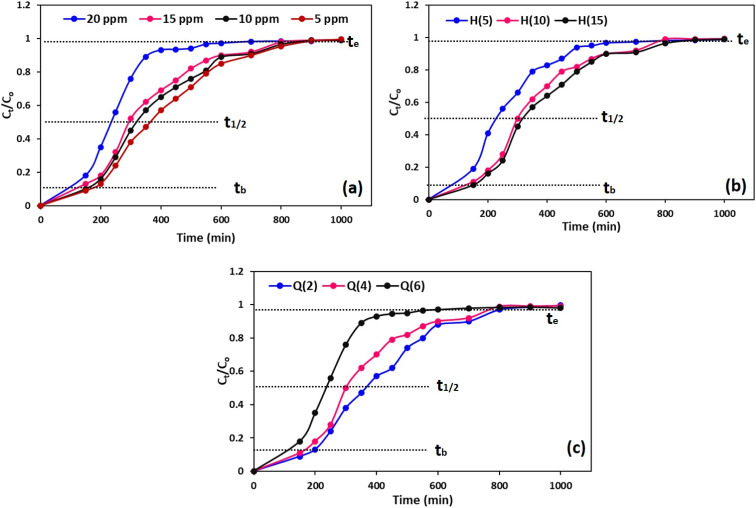
Breakthrough curve at different (a) Cr concentration, (b) bed height, (c) flowrate using NA adsorbent.

To understand and visualise the impact of column parameters (Cr concentration, flow rate, and column height) on breakthrough curves (plots between effluent-to-influent concentration ratios *vs.* time) and investigate different column models, fixed-bed adsorption tests using NA adsorbents were conducted against a simulated Cr solution, as discussed in Section 2.8. Fig. 10a shows the effect of Cr concentration (5, 10,15, and 20 ppm) on the breakthrough curves in the modified sand adsorbent column during 1000 min filtration at pH 4, bed column height (*H*) = 15 cm, and *Q* = 6 min L^−1^. The results revealed that the breakthrough time (*t*_b_), half-time (*t*_1/2_), and exhaustion time (*t*_e_) were highest at the lowest Cr concentration (5 ppm) and a progressive decreasing trend was observed for *t*_b_ from 200 to 150 min, *t*_1/2_ from 400 to 250 min and *t*_e_ from 800 to 680 min with increasing metal ion concentration from 5 to 20 ppm as expected attributed to rapid uptake of contaminants at the cost of early bed saturation due to reduced residence time.^12,55^ Thus, it can be said that the extended breakthrough and exhaustion times at lower concentrations are linked to a greater availability of active sites for the Cr ion, thereby promoting faster adsorption kinetics.^2,21,24^ Similarly, variation of bed height on performance was studied by varying the bed height effect (5, 10, and 15 cm) at pH = 4, *Q* = 5 min L^−1^, Cr concentration = 5 ppm, and results are illustrated in Fig. 10b. Results depict a consistent increase in breakthrough parameters such as increase in *t*_b_ (from 150 to 220 min), *t*_1/2_ (from 250 to 350 min), and *t*_e_ (from 600 to 800 min) with increasing bed height from 5 to 15 cm, attributed to the increased adsorbent amount, thereby increasing the number of active binding sites, which eventually enhanced the contact time between Cr and adsorbent due to a longer path length.^55,56^Fig. 10c depicts the impact of flow rate (2, 4, and 6 mL min^−1^) on the performance of the column at pH = 4, Cr concentration = 5 ppm, *H* = 15 cm. Herein, also *t*_b_, *t*_1/2,_ and *t*_e_ consistently decreased with increasing flow rates from 2 mL min^−1^ to 6 mL min^−1^ because the fast flow rate did not provide sufficient time of contact between Cr and the active adsorption sites. These trends for breakthrough, half, and exhaustion times with respect to adsorbate concentration, bed height, and flow rate are consistent with previous findings.^21,54^

### 3.7 Breakthrough models

**Fig. 11:**
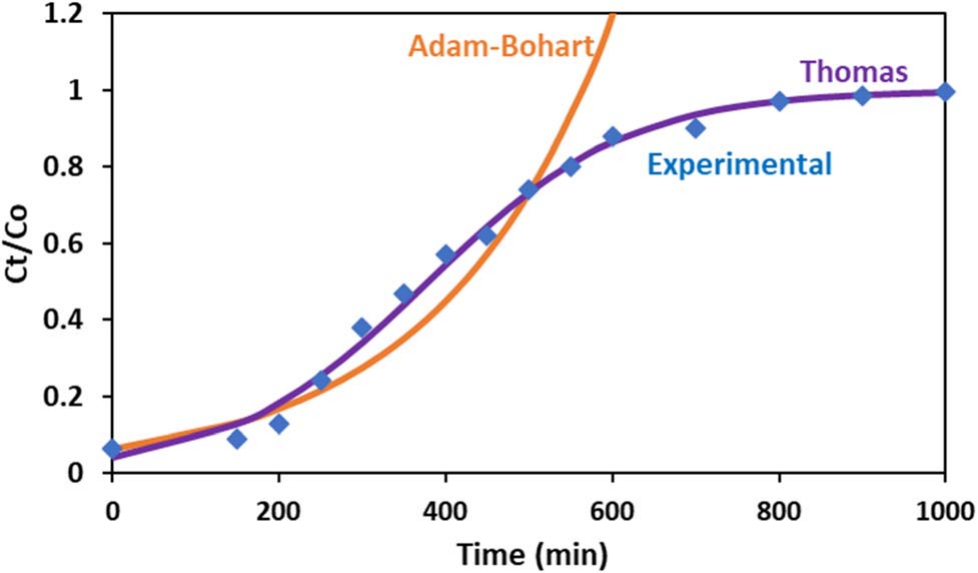
Column kinetic models fitting.

Under optimum conditions (pH = 4, Cr concentration = 5 ppm, *H* = 15 cm, *Q* = 2 min L^−1^), the NA adsorbent/sand column was fitted to the Adams–Bohart and Thomas models, and the corresponding plots are depicted in Fig. 11. The graph clearly depicts Adams–Bohart model were in line with the experimental data till the point when Cr concentration in permeate becomes equal to concentration in feed (*C*_*t*_/*C*_o_ = 1). In contrast, Thomas' model was aligned with experimental data even beyond the breakthrough point. Evaluated kinetic models' parameters suggest that the Thomas model exhibited a superior fit than the Adams–Bohart model, as indicated by the regression coefficient values (*R*^2^) of 0.9873 for the Thomas model and 0.9294 for the Adams–Bohart model. The value of *k*_TH_ in the range of 0.049 L h^−1^ mg^−1^ predicts that the column is kinetically favourable.^25^ Additionally, the strong model correlation supports that Cr(vi) adsorption mechanism follows the Langmuir isotherm and PSO adsorption kinetics and does not account for axial dispersion.^54^ In conclusion, these breakthrough models suggest that the mechanism of Cr adsorption by the NA/sand column was primarily due to mass transfer process (intraparticle diffusion, bulk diffusion, film diffusion) along with adsorption attachment.^25,54^ Comparable findings were reported by ref. 21 for heavy metal removal using a PHW-modified sand column.

### 3.8 Adsorption column study without sand (pure NA only)

**Fig. 12:**
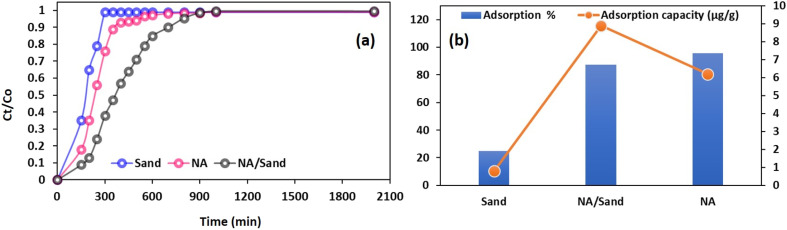
(a) Breakthrough curves for Cr removal, (b) comparison between the performance of sand, NA, and NA/sand column.

To assess the advantages of the NA/sand column over the standalone NA column and the Sand column, a comparative analysis is carried out, and the corresponding results are illustrated in Fig. 12(a and b) under the same conditions that yielded optimal performance for the NA/sand column in terms of Cr removal. For better understanding, the filtration profiles of sand and modified sand (NA/sand) are also displayed in Fig. 12a. Curves depict the exhaustion time for the sand column, NA column, and NA/sand column, approximately 240 min, 400 min, and above 800 min, respectively. The lowest exhaustion time in the sand column is attributed to the high flow rate compared to the other configurations.^24^ As expected, the NA column showed a slightly higher Cr removal efficiency (95.8%) compared to the NA/sand column (87.25%) (Fig. 12b), but it demonstrated a lower adsorption capacity due to the higher mass of NA used. Additionally, the permeate from the NA column exhibited colouration that may be attributed to the leaching of fine NA particles into the permeate, an issue not observed in the NA/sand column.^21,53^ Therefore, the modified NA/sand column is preferred more effective and practical over the other two configurations. Similar observations were also made by Ganji *et al.*, 2024 using sand-modified pistachio hull waste (PHW) for removing different metal ions.^23^ Lastly, to assess the benefits of the proposed method, a comparative study is conducted using published literature and is compiled in Table 7. The table indicates that the NA adsorbent is a promising adsorbent with the potential to convert toxic Cr(vi) to Cr(iii) in both batch and column modes, which is not the case with other adsorbent materials.

**Table 7:** Comparative study of NA adsorbent with other adsorbents in published literature

Adsorbent	Target pollutant (removal%/adsorption capacity)	Treatment technology	Reference
AKX	Adsorption capacity for atrazine 284.8 and 384.0 mg g^−1^	Adsorption (batch & column)	12
PHW	Removal% Ni = 89%	Adsorption (batch & column)	21
MIL/chitosan composite	Removal% tartrazine = 98.40%	Adsorption (batch)	49
Natural clay chitosan nanocomposite	Removal% Ni^2+^ = 81%	Adsorption (batch)	25
	Removal% Cu^2+^ = 80%		
Sand column graphene oxide	Adsorption capacity for Cd(ii) 170.1 mg g^−1^ and Cr(vi) 14.8 mg g^−1^	Adsorption (batch)	24
Fe_3_O_4_-biochar	Removal% plastic particles = 85%	Adsorption (batch)	57
Cyclodextrin–chitosan/GO	Adsorption capacity for Cr(vi) 67.66 mg g^−1^	Adsorption (batch)	45
Graphene oxide/ferric hydroxide	Adsorption capacity for As(iii) 23.78 mg g^−1^	Adsorption (batch)	58
A-WML/TiO_2_ (NA adsorbent)	Adsorption efficiency 94.23% (batch)	Adsorption + photocatalysis (batch & column)	Present study
	Photocatalytic reduction approx. 81% (batch)		
	Bed exhaustion time increases to 800 min @ pH = 4, NPs loading = 3wt%, *H* = 15 cm, flow rate 2 mL min^−1^		

## 4. Limitations of the research and future research directions

A-WML/TiO_2_ nano-adsorbent demonstrated promising Cr(vi) remediation in both batch and sand-mixed fixed-bed column systems; however, several limitations restrict its immediate application in real-world settings. First, the experiments were conducted using synthetic Cr(vi) solutions, which do not fully replicate the complexity of industrial wastewater containing co-ions, organic matter, fluctuating pH, and competing redox reactions. The column trials were performed at a laboratory scale using a controlled feed and UV/sunlight exposure; however, long-term outdoor variability in solar intensity, water turbidity, and flow disturbances may influence photocatalytic–adsorptive performance. Although regeneration was feasible for five cycles, the potential for nanoparticle leaching into the effluent was not quantified, raising concerns regarding secondary environmental risks and compliance with regulatory nanoparticle release limits. The acid-activation and TiO_2_ modification procedures increase processing complexity compared to the use of pristine biomass, which may impact techno-economic feasibility when scaling. The breakthrough time enhancements from 400 to 800 minutes were evaluated under a fixed pH and bed design; thus, the scalability to large hydraulic loads and continuous operations needs further substantiation. Additionally, only Cr(vi) was investigated, whereas industrial effluents often contain multicomponent metal mixtures whose synergistic or inhibitory interactions may alter removal behaviour. Therefore, further advanced engineering and environmental risk assessments are essential before commercialisation.

Future research work should expand testing to real tannery, plating, and dye-industry effluents to assess selectivity and durability in multicomponent environments with variable pH, dissolved solids, anions (SO_4_^2−^, NO_3_^−^), and natural organic matter. Pilot-scale continuous filtration units integrated with solar-driven photocatalytic enhancement should be designed to validate long-term operational stability and hydraulic loading behaviour. The mechanistic investigations, focusing on electron transfer pathways, the formation of intermediate Cr species, and interactions between TiO_2_ anatase and rutile phases, will deepen the understanding of synergistic reactions. Quantifying TiO_2_ leaching, biomass biodegradation, antibacterial functionality, and ecological safety is crucial for establishing environmental compliance. Furthermore, optimisation of nanoparticle loading, bed depth, and hybrid process integration (membrane filtration/biological polishing/advanced oxidation) can increase throughput and energy efficiency. Life-cycle analysis and techno-economic projections should be conducted to compare against commercial adsorbents and justify industrial adoption. Additionally, extending applicability to other emerging contaminants, including Pb(ii), Cd(ii), Ni(ii), As(V/III), dyes, antibiotics, and microplastics, would widen deployment potential in water treatment systems. Harnessing agricultural waste conversion into modular, multifunctional water purification media aligns with circular economy principles and decentralised treatment in rural and industrial clusters.

## 5. Conclusions

This study successfully developed a sustainable nano-adsorbent by incorporating green-synthesised TiO_2_ nanoparticles into acid-modified watermelon leaf biomass (A-WML/TiO_2_) for efficient Cr(vi) removal from surface water under batch and continuous column modes. The synergistic contribution of biomass functionality and TiO_2_ photocatalytic activity significantly enhanced sorption capacity, surface area, and reusability compared to pristine biomass. The nano-adsorbent achieved a maximum Cr(vi) removal efficiency of 94.23% under optimised batch conditions, while also facilitating approximately 81% photocatalytic reduction of Cr(vi) to its less toxic Cr(iii) state. Kinetic and isotherm studies confirmed chemisorption behaviour following pseudo-second-order kinetics and favourable monolayer adsorption under the Langmuir model. Fixed-bed filtration trials further validated the material's scalable applicability by increasing the column exhaustion time from 400 to 800 min, along with an increase in bed height from 5 to 15 cm. Regeneration experiments confirmed the effective reuse of the material over five cycles, with minimal performance loss. Overall, the novel A-WML/TiO_2_ adsorbent integrates waste valorisation, green nanoparticle synthesis, adsorption, and photocatalysis into a single low-cost, environmentally safe treatment platform, demonstrating strong potential for decentralised and continuous heavy-metal removal systems.

## Author contributions

Archana Kushwaha: data collection and curation, experimentation, visualisation, writing – original draft; Zeenat Arif: supervision, administrator, conceptualisation, data curation, investigation, review and editing; Bineeta Singh: supervision, administrator, visualisation, investigation; Ranjeet Kumar Mishra: supervision, administrator, conceptualisation, visualisation, investigation, review and editing.

## Conflicts of interest

There are no conflicts to declare.

## Data Availability

The datasets generated during and/or analysed during the current study are available from the corresponding author upon reasonable request.
